# Maturation of GABAergic Inhibition Promotes Strengthening of Temporally Coherent Inputs among Convergent Pathways

**DOI:** 10.1371/journal.pcbi.1000797

**Published:** 2010-06-03

**Authors:** Sandra J. Kuhlman, Jiangteng Lu, Matthew S. Lazarus, Z. Josh Huang

**Affiliations:** Cold Spring Harbor Laboratory, Cold Spring Harbor, New York, United States of America; RIKEN Brain Science Institute, Japan

## Abstract

Spike-timing-dependent plasticity (STDP), a form of Hebbian plasticity, is inherently stabilizing. Whether and how GABAergic inhibition influences STDP is not well understood. Using a model neuron driven by converging inputs modifiable by STDP, we determined that a sufficient level of inhibition was critical to ensure that temporal coherence (correlation among presynaptic spike times) of synaptic inputs, rather than initial strength or number of inputs within a pathway, controlled postsynaptic spike timing. Inhibition exerted this effect by preferentially reducing synaptic efficacy, the ability of inputs to evoke postsynaptic action potentials, of the less coherent inputs. In visual cortical slices, inhibition potently reduced synaptic efficacy at ages during but not before the critical period of ocular dominance (OD) plasticity. Whole-cell recordings revealed that the amplitude of unitary IPSCs from parvalbumin positive (Pv+) interneurons to pyramidal neurons increased during the critical period, while the synaptic decay time-constant decreased. In addition, intrinsic properties of Pv+ interneurons matured, resulting in an increase in instantaneous firing rate. Our results suggest that maturation of inhibition in visual cortex ensures that the temporally coherent inputs (e.g. those from the open eye during monocular deprivation) control postsynaptic spike times of binocular neurons, a prerequisite for Hebbian mechanisms to induce OD plasticity.

## Author Summary

Evidence suggests that maturation of inhibition is required for the development of plasticity to proceed in the visual cortex. However, the mechanisms by which increased inhibition promotes plasticity are not clear. Here we characterized the maturation of synaptic and intrinsic ionic properties of parvalbumin-positive interneurons, a prominent subtype of inhibitory neuron in the cortex. We used a simple integrate-and-fire model to simulate the influence of maturation of inhibition on associative plasticity rules. We simulated two input pathways that converged onto a single postsynaptic neuron. The temporal pattern of activity was constructed differently for the two pathways: one pathway represented visually-driven activity, while the other pathway represented sensory-deprived activity. In mature circuits it is established that postsynaptic cells can select for sensory-driven inputs over deprived inputs, even in the case that deprived inputs have an initial advantage in synaptic size or number. We demonstrated that maturation of inhibition was required for postsynaptic cells to appropriately select sensory-driven patterns of activity when challenged with an opponent pathway of greater size. These results outline a mechanism by which maturation of inhibition can promote plasticity in the young, a period of development that is characterized by heightened learning.

## Introduction

Association-based Hebbian plasticity is a powerful form of activity-dependent synaptic modification capable of shaping the response properties of neurons during development, and is a proposed substrate for experience-dependent learning [1]–[3]. However, associative forms of plasticity by themselves are destabilizing and must be constrained for circuit activity to remain balanced [4]–[6]. Studies from various preparations demonstrate that both the magnitude and direction of synaptic modification is dependent on the relative timing between pre- and postsynaptic spike events [7]–[13]. Modeling studies indicate that such spike-timing-dependent plasticity (STDP) is inherently stabilizing and competitive because presynaptic inputs that consistently drive postsynaptic spike events ultimately dominate to control postsynaptic spike timing, at the expense of those inputs that are less effective in bringing the postsynaptic cells to threshold. Thus, the net excitatory input onto a given postsynaptic neuron is constant due to re-organization of the synaptic weight distribution [14]. It has been demonstrated that cortical synapses can be modified by STDP rules *in vivo* in response to visual [15]–[18] and whisker [19] stimulation. Importantly, these studies demonstrate that the temporal precision of spike times required for STDP can be propagated from the periphery to the level of the cortex. Thus, STDP may contribute to maintaining circuit stability during experience-dependent plasticity. In the present study we consider how the stabilizing properties of STDP influence experience-dependent plasticity during postnatal development in the visual cortex.

According to the STDP rule, factors that enhance the probability of a given synapse to evoke a postsynaptic action potential, defined as synaptic efficacy [20], will lead to its strengthening. For example, temporal clustering of different inputs is an effective means of increasing synaptic efficacy because inputs that arrive in a temporally coherent group are more likely to summate and bring the postsynaptic neuron to spike threshold. Through such cooperation, a cluster of synapses can grow stronger, while weakening other synapses that are not part of the cluster [21], [22]. Here we use the term *temporal coherence* to refer to the degree of temporal clustering among presynaptic spike times and operationally define it as the width of the cross-correlogram peak among pairs of spike trains within a pathway. In addition to temporal coherence, the initial synaptic strength (ISS) of a synapse is also a major determinant of its impact on postsynaptic spiking [23], [24]. Strong synapses have an advantage among converging inputs, because they are more likely to drive postsynaptic spiking and thus their spike times are likely to fall within the potentiation window of STDP. Indeed, in the developing visual system of the tadpole, Zhang et. al. (1998) demonstrated that synaptic strength is capable of conferring a competitive advantage during STDP-mediated synaptic re-organization. This raises the question of whether and how initially strong synapses can be weakened, and which parameter, temporal coherence or initial synaptic strength, determines the outcome of synaptic competition.

This issue is relevant to ocular dominance (OD) plasticity in the developing primary visual cortex (V1). During a postnatal critical period, monocular deprivation (MD) causes binocular neurons in the primary visual cortex to shift their responsiveness towards open-eye inputs via a Hebbian-based process [1], [25]. Importantly, monocular blurring using an overcorrecting contact lens, which distorts but does not eliminate vision, is equally effective in inducing a shift in ocular dominance [26]. These results suggest that it is the pattern of visual input, likely manifested as the temporal correlation of retinal afferent activity, that drives plasticity [27], [28]. In rodents, the majority of neurons in the binocular visual cortex are normally dominated by inputs from the contralateral eye, yet closure of this eye during the critical period results in the weakening of its inputs and strengthening of the ipsilateral, open eye inputs. Therefore, temporally coherent inputs are able to overcome the initially strong but less coherent inputs and eventually dominate in driving binocular neuron responses. However, the conditions and cellular mechanisms that confer an advantage to ipsilateral inputs are not well understood.

GABAergic inhibition potently influences input summation, which is required for spike generation, by restricting the temporal window over which inputs are able to effectively cooperate [29]–[31]. Intuitively, increasing the strength of GABAergic inhibition would seem a good candidate for shifting the control of postsynaptic spiking to inputs with a higher temporal coherence versus inputs with a higher synaptic strength. In addition, GABAergic inhibition develops in a protracted postnatal period [32]–[34], and this protracted development was shown to regulate the timing of the critical period for OD plasticity [35]–[40]. We therefore hypothesize that the developmental increase of inhibition in V1, by biasing the control of postsynaptic spiking, ensures that Hebbian plasticity mechanisms are engaged during MD to strengthen the temporally coherent inputs over those with higher initial synaptic strength.

Here we combine modeling and experimental approaches to examine the role of GABAergic inhibition in promoting the selective strengthening of temporally coherent inputs in the context of OD plasticity. We characterized the maturation of a major class of GABAergic interneuron in rodent visual cortex and found that both synaptic and intrinsic properties of Pv+ interneurons changed dramatically during the critical period of OD plasticity. Using a simple integrate-and-fire neuron model driven by inputs modifiable by STDP, we determined that a sufficient amplitude of synaptic inhibition along with an increase in gain of GABAergic neuron spike output was required to ensure that temporal coherence, rather than initial synaptic strength, controlled postsynaptic spike timing. Inhibition exerted this effect by preferentially reducing the synaptic efficacy of the less coherent inputs. The modeling results predict that the developmental increase in inhibition should decrease synaptic efficacy during the critical period. Indeed, using acute cortical slices of visual cortex, we found that stimulus-evoked synaptic inhibition potently reduced synaptic efficacy at the peak of but not prior to the onset of the critical period.

## Results

Our modeling and experimental studies were driven by a desire to understand the role of GABAergic inhibition in regulating synaptic plasticity, especially ocular dominance (OD) plasticity in the visual cortex in response to contralateral eye deprivation. We first examined whether synapses in the mouse primary visual cortex are modifiable by STDP.

### Layer 2/3 synapses in V1 are modified by STDP prior to and at the peak of the critical period of OD plasticity

**Figure 1:**
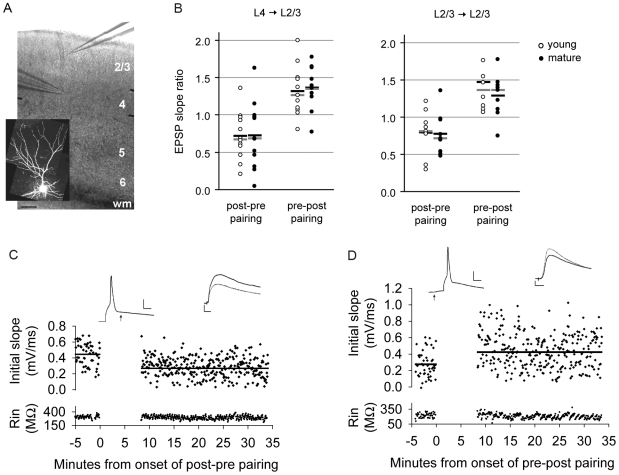
STDP is inducible in mouse primary visual cortex before and after the onset of the critical period of OD plasticity. (A) Position of field stimulation electrode in layer 4 and whole-cell recording electrode in layer 2/3 in V1, scale bar: 100 microns. WM: white matter. Inset: an alexa-594-filled L2/3 pyramidal neuron. (B) Mean (black bar) and median (gray bar) EPSP slope ratios for young (open circle, LTD n = 12, LTP n = 11) and mature (closed circle, LTD n = 12, LTP n = 11) age groups at vertical layer 4→ layer 2/3 connections (left) and layer 2/3→ 2/3 connections (right). (C) Example of an individual cell in which the EPSP followed the action potential by 9 ms, age = P17. (D) Example of an individual cell in which the EPSP preceded the action potential by 9 ms, age = P17, scale bar 25 mV, 5 ms. Baseline traces are represented by solid lines, post-pairing traces are represented by dashed lines, scale bar: 0.5 mV, 10 ms.

Precocious OD plasticity can be triggered by enhancing GABAergic transmission within V1 [36], [39], [40], suggesting that the machinery for OD plasticity is operational before its natural onset, but lies dormant until local GABAergic inhibitory circuits mature. To examine whether the basic mechanisms for synaptic plasticity are present at glutamatergic connections prior to the onset of OD plasticity, we compared the ability to induce STDP at layer 2/3 synapses in acute slices of V1 prior to the onset (postnatal day 16–18) and at the peak (P26–30) of OD plasticity. Long-term synaptic depression (LTD) or potentiation (LTP) was induced using a STDP protocol. Postsynaptic action potentials were evoked by current injection from the recording electrode, bypassing the need for inputs to summate to bring the cell to spike threshold. Whole-cell current-clamp recordings were made from layer 2/3 pyramids (Figure 1A), in the presence of 10 µM picrotoxin. EPSPs were continuously evoked at a frequency of 0.2 Hz throughout the experiment from a field electrode placed in layer 4. EPSPs were monitored for a baseline period of 5 minutes, then paired 100 times with an action potential (AP), and further monitored for 20–45 minutes. To induce LTD, the AP was timed to precede the EPSP by 9+/−2 ms. To induce LTP, the AP was timed to follow the EPSP by 9+/−2 ms.

An example of LTD induction from a P17 slice is shown in Figure 1C. The average initial slope of the EPSP during the baseline period was 0.44 mV/ms. Following the AP-EPSP pairing protocol, the average initial slope of the EPSP decreased to 0.27 mV/ms. We calculated the EPSP slope ratio (EPSP slope post-pairing/EPSP slope pre-pairing) to compare plasticity across slices and ages. In both young and mature slices there was a significant reduction of the mean EPSP slope ratio following the LTD protocol. The mean EPSP slope ratio was 0.72+/−0.09 in young slices (p<0.05, paired t-test, n = 12); and 0.72+/−0.13 in mature slices (p<0.05, paired t-test, n = 12). There was no significant difference between the two ages (Figure 1B), determined using either a t-test (p = 0.97) or a Kolmogorov-Smirnov (KS) test (p = 0.99), which is sensitive to differences in data distribution as well as the mean.

An example of LTP is shown in Figure 1d. The baseline EPSP slope was 0.28 mV/ms. Following the AP-EPSP pairing protocol, the EPSP slope increased to 0.42 mV/ms. Similar to the LTD protocol, the LTP protocol induced significant plasticity at both ages. The mean EPSP slope ratio was 1.32+/−0.10 in young slices (p<0.05, paired t-test, n = 11), and was 1.36+/−0.09 in mature slices (p<0.05, paired t-test, n = 11). There was no significant difference between the two ages (t-test, p = 0.76; KS, p = 0.81).

We also compared the ability to induce STDP at local recurrent connections within layer 2/3 in the two age groups (Figure 1B). To stimulate local recurrent connections, the field electrode was placed laterally within 50 microns of the recorded cell. Similar to layer4→layer2/3 connections, we found that the activated synapses were modifiable by STDP in both young and mature slices. In response to the LTD protocol the mean EPSP slope ratio was 0.79+/−0.09 in young slices (p<0.05, Wilcoxon signed rank, n = 10), and was 0.78+/−0.09 in mature slices (p<0.05, Wilcoxon signed rank, n = 9). There was no significant difference between the two ages (KS, p = 0.25). In response to the LTP protocol the mean EPSP slope ratio was 1.47+/−0.16 in young slices (p<0.05, Wilcoxon signed rank, n = 8), and 1.29+/−0.10 in mature slices (p<0.05, Wilcoxon signed rank, n = 9). There was no significant difference between the two ages (KS, p = 0.90).

Therefore, by bypassing the requirement for input summation, we demonstrated that STDP was similarly induced at layer 4→ layer 2/3 connections as well as local recurrent connections in mouse primary visual cortex both prior to the onset and at the peak of the critical period of OD plasticity. Our results, along with others [41], raise the possibility that the ability to induce plasticity at glutamatergic synapses may not be a primary factor in determining the onset of OD plasticity.

### Maturation of GABAergic synaptic and intrinsic properties in primary visual cortex

**Figure 2:**
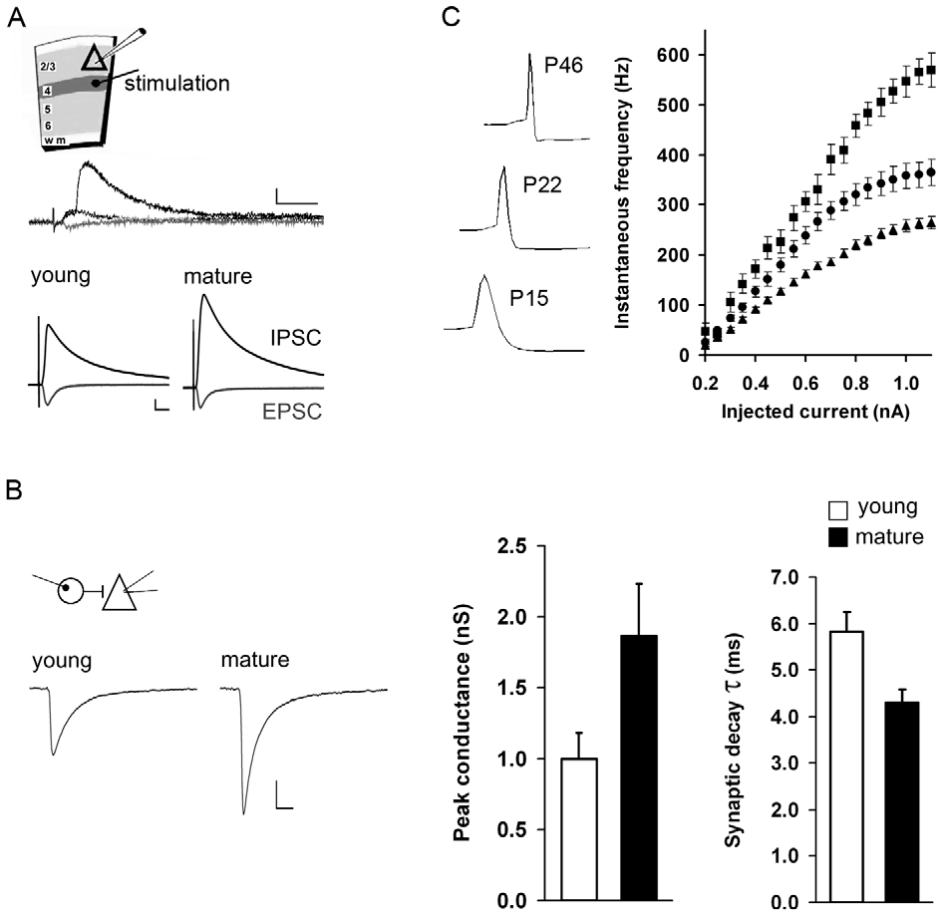
Maturation of GABAergic synaptic and intrinsic properties in primary visual cortex. (A) Stimulus recording configuration for determining the maximal IPSC amplitude prior to (young) and during (mature) the critical period. Upper trace, example of disynaptic IPSC activity in a layer 2/3 pyramidal neuron in response to stimulation of layer 4, 15 µA intensity. IPSC responses are from two separate trials (black), EPSC (gray); scale bar: 50 pA, 10 ms. Lower traces, amplitude of isolated monosynaptic AMPA-mediated current (EPSC, recorded at the empirically determined GABA_A_ reversal potential, gray) and compound GABA_A_-mediated current which included both monosynaptic and polysynaptic IPSCs (recorded at 0 mV, black) in layer 2/3 pyramidal neurons in response to stimulation of layer 4. Stimulus input- output curves for a range of stimulation intensities (10–80 µA) were generated (see Figure S1). The average current from all trials in which a 350±50 pA EPSC was elicited is shown here. Young: n = 9 (of 14 cells) fulfilled this criteria, mature: n = 8 (of 13 cell) fulfilled this criteria. The normalized IPSC charge (nA*ms) shown here increased 1.7-fold with age (young, 36.5+/−3.6; mature, 62.0+/−9.8 pC, t-test p<0.05). GABA_B_ and NMDA-mediated currents were blocked. Scale bar: 250 pA, 10 ms. (B) Unitary IPSCs recorded in layer 2/3 pyramidal neurons in response to stimulation of single Pv+ basket interneurons prior to the critical period (n = 14), and during the critical period (n = 19). Left, averaged current responses across all cells. The average IPSC amplitude increased 1.8-fold with age, while the average synaptic decay time-constant decreased roughly 25%, from 5.8 to 4.3 ms. See Table 1 for statistics. (C) Input/output curves of instantaneous firing frequency of Pv+ basket interneurons prior to (n = 27, p14–15, triangles), during (n = 21, p19–23, circles), and at the end of the critical period (n = 5, p44–46, squares). Maximal spike output in response to the same input increased by greater than 2-fold during the course of the critical period (right). There was a corresponding decrease in spike half-width during development, p14–15 (0.96+/−0.18 ms), p19–23 (0.56+/−0.14 ms), p44–46 (0.23+/−0.03 ms), example voltage traces of spike shape shown on the left.

To examine the changes of GABAergic inhibition onto V1 pyramidal neurons during the critical period, we assayed the maximal inhibitory input onto layer 2/3 pyramids at two developmental ages, just prior to the onset (young) and during (mature) the critical period. Postsynaptic responses in layer 2/3 pyramidal neurons were recorded in response to stimulation of layer 4, which evoked a mixed excitatory-inhibitory response (Figure 2A). Similar to previous reports [42], we found that inhibitory drive increased with age relative to excitatory drive (Figure 2A), and that the maximal inhibitory charge significantly increased with age, while the maximal excitatory charge was stable (Table 1). Parvalbumin-containing (Pv+) basket cells make up ∼50% of GABAergic interneurons in rodent V1, and it has been shown that there is a ∼2-fold increase in the number of Pv+ basket presynaptic terminals surrounding pyramidal somata during the critical period [34]. To determine if there was a corresponding increase in synaptic function, we recorded from synaptically connected Pv+ interneuron to pyramidal neuron pairs in layer 2/3. Pv+ interneurons were recorded using either BAC transgenic mice in which the Pv promoter drives GFP [43] or Pv-cre mice [44] injected with a recombinant adeno-associated virus that expresses GFP specifically in Pv+ basket cells [45]. We found that peak inhibitory synaptic conductance increased by 1.8-fold during the critical period compared to prior to the onset of critical period, while there was a 25% decrease in the synaptic decay time-constant (Figure 2B, Table 1). In contrast to inhibitory connections, paired recording of pyramidal neurons revealed that the peak excitatory synaptic conductance was similar between young (n = 10) and mature (n = 10) layer 2/3 connections (0.17+/−0.09 and 0.23+/−0.14 nS, respectively).

**Table 1:** Synaptic properties of layer 2/3 neurons of V1.

L4 fld stm → L2/3 Pyr wc recording	Inhibitory current		Excitatory current	
	young	mature	young	mature
maximal charge (pC, nA* ms)	63.20+/−31.97	147.83+/−75.82 *	0.37+/−0.17	0.44+/−0.23

L2/3 → L2/3 wc paired recording	Pv+ → Pyr		Pyr → Pyr	
	young	mature	young	mature
peak conductance (nS)	0.98+/−0.69	1.80+/−1.62 *	0.17+/−0.09	0.23+/−0.14
decay time-constant (ms)	5.84+/−1.62	4.30+/−1.13 *	2.48+/−0.67	2.56+/−0.59

Synaptic currents were measured by whole-cell (wc) voltage-clamp recordings of layer (L) 2/3 pyramidal neurons and stimulating presynaptic inputs. Maximal charge was measured using the L4 field stimulation (fld stm) configuration shown in Figure 2A (inhibitory: young, n = 13, mature, n = 12; excitatory: young, n = 13, mature, n = 12). For paired recordings, either presynaptic parvalbumin interneurons (Pv+; young, n = 14; mature, n = 19) or presynaptic pyramidal (Pyr) neurons (young, n = 10; mature, n = 10) were stimulated as indicated. +/− std. dev.

In addition to synaptic properties, we characterized the intrinsic properties of Pv+ interneurons (Table 2) and found that the current input/spike out curve shifted during the critical period: for the same stimulus input, the number of output spikes was greater during the critical period compared to that prior to the onset of the critical period. Thus, the gain of Pv+ interneurons increased during the critical period. In addition, there was a corresponding decrease in spike half-width (Figure 2C).

**Table 2:** Intrinsic properties of layer 2/3 neurons of V1.

	Pv+		Pyr	
	young	mature	young	mature
Input resistance (mΩ)	160.0+/−74.3	130.8+/−19.5	269.4+/−60.9	150.1+/−36.9 *
Membrane time-constant (ms)	6.13+/−3.4	3.93+/−1.5 *	25.31+/−7.8	14.68+/−4.9 *
Spike half-width (ms)	0.96+/−0.18	0.56+/−0.14 *	1.13+/−0.11	0.97+/−0.15 *

Intrinsic properties were measured by whole-cell current-clamp recordings of Pv+ (young, n = 14; mature, n = 15) or Pyr neurons (young, n = 15; mature, n = 16). +/− std. dev.

These electrophysiological results are summarized in Tables 1 and 2. Relative to excitatory connections, synaptic inhibition significantly matured with age. These results do not exclude the possibility that there are subtle developmental changes in synaptic excitation. In contrast to synaptic properties, the intrinsic properties of pyramidal cells, including input resistant, changed with age, as previously reported [46].

### A simple point conductance model driven by two convergent input pathways modifiable by STDP

Modeling studies have demonstrated that in response to a change in the temporal pattern of presynaptic spike times, STDP implemented in its most basic form, re-organizes the population of synapses converging onto a postsynaptic neuron such that the most coherent inputs are strengthened, while the remaining synapses are weakened [22]. The outcome of STDP driven re-organization is that the net excitatory drive across a population of synapses converging onto a single postsynaptic neuron is stabilized and a subset of presynaptic inputs controls postsynaptic spike timing. Here we extended the Song-Miller-Abbott (2000) model to include two distinct convergent input pathways, and tested the effects of altering presynaptic spike times within a pathway on the ability of the pathway to control postsynaptic spike timing, across a range of inhibitory levels. It has been observed that strong synapses can in some situations undergo less potentiation than weak synapses [47], therefore we also ran the simulation in a weight-dependent mode in which the amount of potentiation was inversely related to synaptic size.

**Figure 3:**
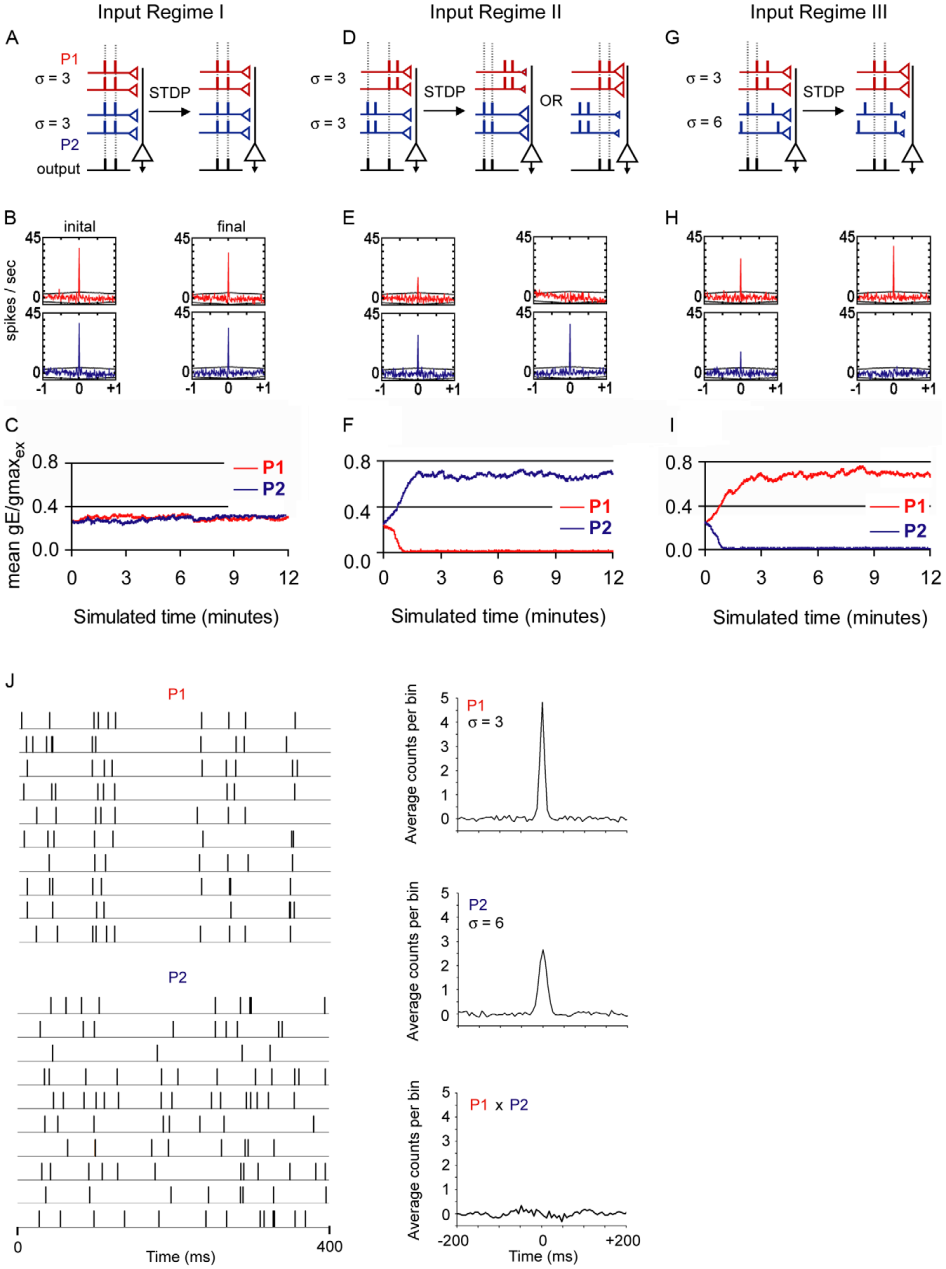
A simple IAF model neuron driven by two convergent input pathways subject to the STDP rule. The synaptic weight re-organization following iterations of the STDP rule and the impact on correlations between pre- and postsynaptic spike times in response to 3 input regimes are summarized schematically in the top row (A, D, G; also see text). Large black triangles represent postsynaptic pyramidal neurons. Red line and small triangle represent input pathway 1 (P1). Blue line and small triangle represent input pathway 2 (P2). The size of red and blue triangles symbolize synaptic weights. Short vertical lines represent spike times in P1 (red), P2 (blue), and postsynaptic pyramidal neurons (black). Vertical dashed lines highlight the presence of significant cross-correlation between pre- and postsynaptic spike times. σ represents the temporal jitter in spike times of inputs within a pathway. Note that in Regime III (G–I), inputs in P2 are not aligned, denoting higher spike jitter (σ = 6) compared to those in P1 (σ = 3). Middle row (B,E,H), cross-correlogram plots of example trials depicting the cross-correlation of an individual presynaptic spike train from either P1 (red) or P2 (blue) versus the postsynaptic spike train, calculated for the initial 50 seconds (left column) and the final 50 seconds (right column) of the simulation, in the case of (F), a trial in which P2 dominated is shown. Units for the x-axis are ms. Out of the 40 possible presynaptic spike trains, the presynaptic spike train with the highest peak correlation value is shown. Peak cross-correlogram values for all 40 presynaptic spike trains for P1 and P2 are shown in Figure S2. Note that in all three regimes, both P1 and P2 provide threshold input and contribute to postsynaptic spike events early in the simulation. Bottom row (C,F,I), mean synaptic weight of P1 and P2 over the course of the simulation (same example trials as in B,E,H), in units of conductance (gE) divided by the maximal excitatory synaptic conductance (gmax_ex_). In all cases, gI/gmax_ex_ = 0.264. (J) Left, raster plot of presynaptic spikes used in Regime III simulation, each row represents a single spike train (10 of 40 shown). Right, cross-correlation between two randomly selected spike trains from P1(top), P2(middle), and between the two pathways (bottom). Bin size was 5 ms.

We used an integrate-and-fire model neuron driven by 2 presynaptic pathways that each contained 40 synaptic inputs. As in Song et. al. (2000), a function *F*(Δ*t*) determined the amount of excitatory synaptic modification arising from a single pair of pre- and postsynaptic spikes separated by a time Δ*t* (Methods). Excitatory synaptic conductance was not allowed to exceed a maximum value gmax_ex_. If the modification function pushed the synaptic weight past the gmax_ex_ value, the weight was reset to the appropriate limiting value. The maximum amount of modification for a single pre- and postsynaptic spike pair corresponded to a 0.5% change of gmax_ex_. This function provides a reasonable approximation of the dependence of synaptic modification on spike timing observed experimentally, and makes no assumptions regarding the mechanism(s) of STDP. The model neuron also received inhibitory conductance: each excitatory conductance was followed by an inhibitory conductance of fixed amplitude with a delay randomly varying between 4 to 10 ms. For the simulations in Figure 3, the ratio of the amplitude of inhibitory conductance over gmax_ex_ (gI/gmax_ex_) was 0.264, and the average initial synaptic strength (ISS), was the same for both pathways, set to 25% of gmax_ex_. This amount of inhibition was sufficient to maintain the postsynaptic neuron in a balanced mode, defined by an excitatory-inhibitory ratio of 1.1–1.2 at the threshold for action potential generation [14].

A minimal number of parameters were used to generate presynaptic spike trains (see Methods). We then altered two of these parameters to modify the temporal relationships among inputs within and between the two pathways. First, we altered the *temporal correlation* between pathway 1 (P1) and pathway 2 (P2), defined as whether or not the two pathways share coincident presynaptic spike times. Second, we altered the *temporal coherence* (1/σ) among inputs within a single pathway, which refers to the degree of temporal clustering of spike times; the value σ represents the temporal jitter (ms) of presynaptic spike times (Figure 3).

In our baseline condition, Input Regime I (Figure 3A–C), presynaptic spike times were correlated between P1 and P2, and presynaptic spike times within the two pathways had the same degree of high temporal coherence (σ_P1_ = 3, σ_P2_ = 3). This input regime represents features of a normal binocular neuron, which receives converging and correlated inputs from the two eye pathways, and activity within each pathway displays high temporal coherence driven by the same visual stimulus. Ten independent trials of the simulation were run. Cross-correlation analysis of individual presynaptic spike trains versus the postsynaptic spike train demonstrated that each pathway was capable of driving postsynaptic events during the initial 50 seconds of simulated time, and also in the final phase of the simulation (Figure 3B). Cross-correlation results are schematized in Figure 3a. As expected for this input regime, the mean synaptic weight for each pathway was unchanged during the course of the simulation (Figure 3C), both pathways maintained the ability to control postsynaptic spike timing throughout the simulation, as indicated by the dashed lines in Figure 3A.

In Input Regime II (Figure 3D–F), presynaptic spike times between the two pathways were de-correlated, while the high degree of temporal coherence within each pathway (σ_P1_ = 3, σ_P2_ = 3) was preserved. This input regime represents features of a binocular neuron during strabismus, in which inputs from the two eyes are de-correlated and spike times within each eye-specific pathway display high temporal coherence. Ten independent trials of the simulation were run. The mean synaptic weight of one pathway strengthened, at the expense of the opposing pathway, such that the total excitatory driving force was maintained and the firing rate of the postsynaptic neuron was stable. The outcome of which pathway, P1 or P2, dominated occurred at chance level.

For Input Regime III (Figure 3G–J), in addition to temporal de-correlation between the pathways, temporal coherence was reduced in Pathway2 (σ_P1_ = 3, σ_P2_ = 6, a σ ratio of 1∶2). This input regime represents features of a binocular neuron during monocular deprivation, in which the two eye-specific pathways are uncorrelated and the temporal structure of activity differs between the two pathways. The open eye views high-contrast patterns, therefore the open-eye pathway likely has a relatively higher degree of temporal coherence compared to the closed-eye pathway [26]–[28]. The temporal structure of the presynaptic spike trains used in the simulation is shown in Figure 3J. Ten independent trials were run. Pathway1, with higher temporal coherence, always attained the higher mean synaptic weight and emerged to drive spike output. In addition, the spike times of the postsynaptic neuron became controlled by P1 in all trials. Our model thus demonstrates that the stabilizing and competitive properties of STDP first described by Song et. al. (2000), also apply to the condition of two independent convergent pathways, and that the pathway with relatively higher temporal coherence will dominate in driving postsynaptic spike times when the initial synaptic strength is equal between pathways.

### Initial synaptic strength confers a competitive advantage

In addition to the temporal structure of presynaptic inputs, initial synaptic strength plays a major role in driving postsynaptic spiking and is also likely to contribute to the outcome of STDP-driven re-organization of inputs. Inputs with higher synaptic strength have an advantage because fewer active synapses are required to evoke a postsynaptic action potential. Indeed, at the retinotectal projection in tadpoles, it has been demonstrated that the extent to which a given pathway potentiates in response to asynchronous stimulation of convergent inputs is dependent on initial synaptic strength [9].

**Figure 4:**
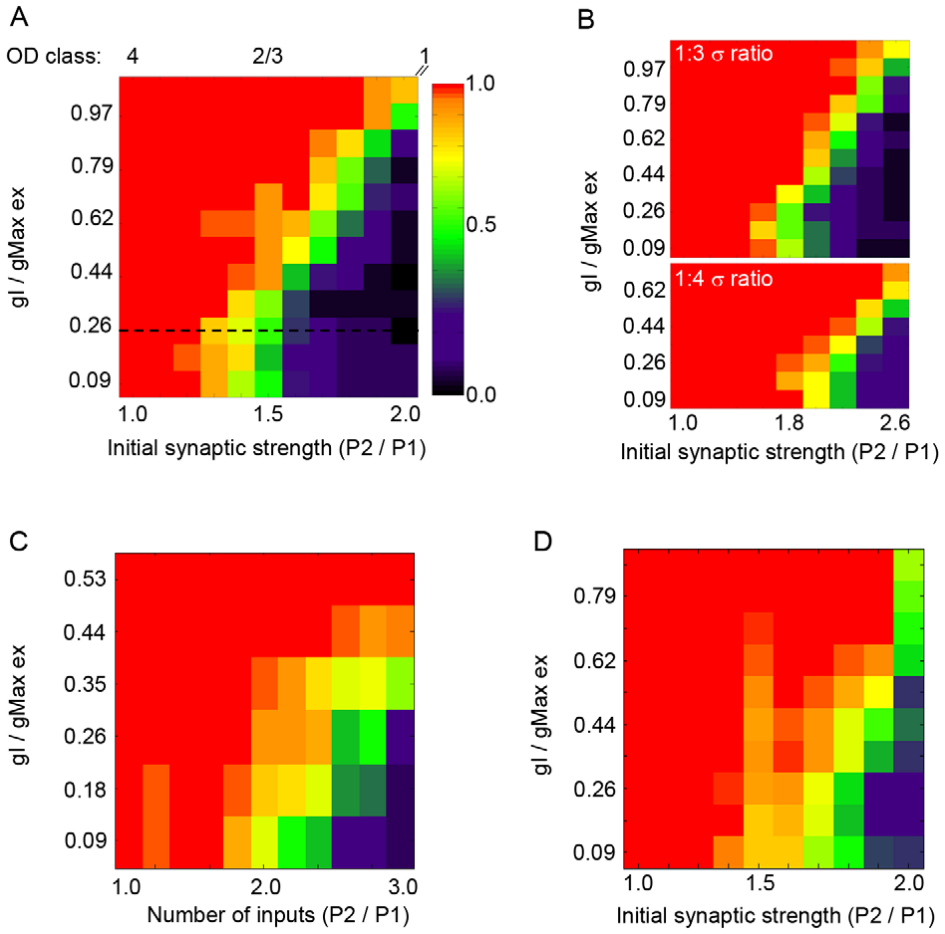
The level of inhibition determines whether temporal coherence or synaptic drive controls postsynaptic spike times in the IAF model. Color shades indicate fraction of trials in which P1 out-competed P2: red = 100%, black = 0%. (A) At the lowest amplitude of inhibition, the range of initial synaptic strength ratios (ISS_P2/P1_) over which the temporally more coherent P1 pathway can compete with the stronger but less coherent P2 pathway is narrow (between 1–1.2) and restricted to a subset of OD class 4 cells. At a high level of inhibition, the range of ISS_P2/P1_ ratios over which P1 successfully out-competed P2 above chance level (0.5) is extended to include the full spectrum of OD class 4 and the majority of class 2/3 cells. In this case, pathway temporal coherence (σ^−1^) between the two pathways was set to a ratio of 1∶2 (P1:P2). (B) The influence of inhibition holds for a range of temporal coherence pathway ratios. (C) Increased inhibition had a similar influence when contralateral bias was implemented as a difference in number of synaptic inputs, rather than initial synaptic strength, P2/P1 ratios ranging from 1 to 3 were tested. (D) The ability of increasing inhibition to provide a competitive advantage to P1 over P2 is maintained in an implementation of weight-dependent STDP. In this case, the amount of potentiation for a given LTP event was inversely related to synaptic strength A_+_ * (1-α), see Methods for more details.

Using Input Regime III (σ ratio of 1∶2), we challenged the ability of P1, the temporally more coherent pathway, to control postsynaptic spike timing by increasing the initial synaptic strength (ISS) of P2. The ISS ratio (P2/P1) was varied from 1.0 to 2.0. Using the same level of inhibition as in Figure 3 (gI/gmax_ex_ = 0.264), we found that the fraction of simulation trials in which P1 controlled postsynaptic spike timing decreased with increasing strength of P2 (Figure 4A, dashed line). When P2 was initially 50% stronger (ISS_P2/P1_ ratio  = 1.5), it dominated in driving postsynaptic spike activity in only half of the trials. Thus when the ISS_P2/P1_ ratio was ≥1.5, P1, the pathway with higher temporal coherence, failed to direct postsynaptic spike timing above chance level. This result, however, formally contradicts with the results of OD plasticity in V1.

The majority of binocular neurons in V1 are not equally driven by the two eye-specific pathways. In rodents, approximately 70% of binocular neurons [25] are characterized as class 2/3 cells, preferentially driven by the contralateral eye inputs. Despite this contralateral bias, contralateral eye closure during the critical period shifts the response properties of class 2/3 cells such that they become dominated by the initially weak, but temporally more coherent ipsilateral eye inputs. Our result in Figure 4A, in which the pathway with higher temporal coherence fails to direct postsynaptic spikes, is thus inconsistent with the OD shift of class2/3 neurons induced by monocular deprivation. In the following section we tested the hypothesis that synaptic inhibition can constrain STDP and promote the selective strengthening of temporally coherent inputs at convergent pathways, even when challenged with inputs of higher initial synaptic strength.

### Inhibition biases STDP to favor temporal coherence over initial synaptic strength

The stabilizing influence of STDP on net excitatory drive is highly dependent on the non-linearity of the spike generation process [22]. Given that synaptic inhibition has been shown to potently influence input summation by restricting the temporal window over which inputs are able to effectively cooperate [29]–[31], we tested if increasing the amplitude of synaptic inhibition in Input Regime III could bias the weaker but temporally more coherent pathway (P1) to control postsynaptic spike timing. The simulation was run at 6–12 different levels of inhibition and the strength of P2 was increased 2 to 3-fold relative to P1. The simulation was run for 30–50 independent trials for each parameter pair, and the same presynaptic spike train was used for each level of inhibition for a given ISS_P2/P1_ ratio (Figure 4A). We found that the range of ISS_P2/P1_ ratios in which P1 out-competed P2 was extended when inhibition was high, and that for a given ISS_P2/P1_ ratio, higher levels of inhibition increasingly biased the outcome of STDP-driven competition to favor P1 over P2 (Figure 4A). For example, in the case that P2 was set to be 50% stronger than P1 (ISS_P2/P1_ = 1.5) and the amplitude of inhibition was set to ≥0.792 gI/gmax_ex_, P1 dominated in 100% of the trials, while at lower levels of inhibition (gI/gmax_ex_ = 0 to 0.264), P2 out-competed P1 in roughly 50% of the trials. We examined a range of relative temporal coherence values and found that increasing inhibition had a similar effect as above in cases that the ISS ratio was sufficiently high to give an advantage to P2 (Figure 4B).

As previously reported [46], we found that the input resistance of layer 2/3 pyramidal cells decreased with age (Table 1). A change in input resistance (R_in_) could potentially influence summation and therefore impact the effect of inhibition in our simulation. However, there was a parallel change in the membrane time-constant (τ_mem_). Given the relationship, τ_mem_ =  R_in_ * whole-cell capacitance, whole-cell capacitance remained stable. In acute slice experiments, values of R_in_ are typically about 30% higher than in vivo studies [48]. We examined a physiologically realistic range of whole-cell capacitance values in our simulation (from 0.125 nF to 0.375 nF) and found that the effect of increasing inhibition was independent of whole-cell capacitance (Figure S3).

In the above simulations we implemented contralateral bias as an increase in initial synaptic strength. However, contralateral bias in vivo could be the result of an increased number of inputs rather than increased synaptic strength (or a combination of the two). Therefore we examined the effect of increasing inhibition across an increasing number of P2 inputs and found that as with synaptic strength, increased inhibition helped to ensure that the temporally coherent pathway out-competed the pathway with an initially stronger synaptic drive (Figure 4C).

**Figure 5:**
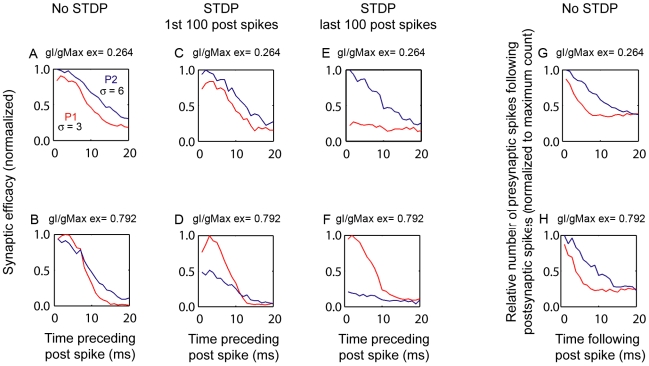
The relative contribution of input pathways with different temporal coherence and initial synaptic strength to postsynaptic spike events is dependent on inhibition. In these simulations, P1 (red, σ = 3) is more coherent than P2 (blue, σ = 6), as in Input Regime III (Figure 3G), but is weaker (ISS_P2/P1_ = 1.5). The relative number of presynaptic spikes in P1 or P2 that preceded postsynaptic spike events was counted in 1 millisecond time bins and plotted as a function of time preceding the postsynaptic spike for two levels of inhibition, low (A,C,E, gI/gmax_ex_ = 0.264) and high (B,D,F, gI/gmax_ex_ = 0.792). The analysis was first done in the absence (A, B) and then in the presence (C–F) of the STDP learning rule, (C–F) are examples of individual STDP simulations trials. At a low level of inhibition, P2 had a slight advantage in all time bins in the absence of STDP (A), and went on to dominate in 50% of the STDP trials; an example of P2 domination shown in (C, E). At a high level of inhibition, P1 had a slight advantage over P2 in contributing to postsynaptic spike events 2–8 ms preceding the postsynaptic spike (B); and this advantage was dramatically magnified by STDP (D,F). (G–H) The total number of presynaptic spikes in P2 that *followed* postsynaptic spike events is greater than that in P1 in the absence of STDP learning rules. The relative number of presynaptic spikes was calculated as in (A–B), except that 1 ms bins counts were made for a time window of 20 ms following postsynaptic spikes.

A likely mechanism by which stronger inhibition biased STDP to favor P1 is that inhibition narrowed the window of input cooperation, thereby preferentially restricting the less coherent inputs in P2 from contributing to postsynaptic spike generation. If this is the case, then the relative ability of P1 synapses to drive a postsynaptic spike event (synaptic efficacy) should increase with inhibition, independent of the STDP learning rule. To examine this possibility, we compared the efficacy of P1 and P2 synapses in a simulation implemented as in Figure 3A, except without applying the STDP learning rule. As expected, increasing the level of inhibition (from gI/gmax_ex_ = 0.264 to 0.729) decreased the relative efficacy of P2 synapses. At a high level of inhibition, P1 synapses had a slight advantage in driving postsynaptic events 2–8 ms preceding the postsynaptic spike (Figure 5, compare A&B), a temporal window for which STDP-mediated potentiation is the strongest. This slight advantage of P1 was robustly magnified when the STDP learning rule was applied in the simulation. Within the first 100 spikes of the simulation, the relative efficacy of P1 synapses increased compared to the no-STDP condition (Figure 5, compare B&D). P1 synapses completely dominated by the end of the simulation (Figure 5, compare D&F). Analysis of synaptic efficacy was also performed on the results from the initial simulations shown in Figure 3 (see Figure S4). Similarly, we found that the pathway having a slight advantage after the first 100 spikes of the STDP simulation would ultimately dominate.

We previously showed that at a low level of inhibition (gI/gmax_ex_ = 0.264), P2 out-competed P1 in 50% of the STDP simulation trials (Figure 4A, ISS_P2/P1_ ratio = 1.5). It was surprising therefore to find that in the absence of STDP, P2 dominated in driving postsynaptic spikes for the full 20 ms time window (Figure 5A, upper left). Given this advantage, P2 would be expected to dominate in 100% of the STDP simulation trials rather than only 50%. The reason that P2 did not dominate in 100% of the trials was that the number of presynaptic spikes occurring *after* each postsynaptic spike was greater for P2 than P1 (Figure 5G), thus leading to more LTD in P2 than in P1 when the STDP learning rule was applied. Importantly, the difference in the number of presynaptic spikes *following* postsynaptic spike events between the two pathways was similar for both low and high levels of inhibition (Figure 5G,H). Therefore, the preferential restriction of P2 inputs (Figure 5 A,B) was due to a specific decrease in their synaptic efficacy rather than a increase of their LTD at higher levels of inhibition.

It has been shown that under some conditions, the amount of synaptic potentiation is dependent on synaptic strength [13], [49], and that this can impact the outcome of STDP [47]. Therefore, we ran the same simulation shown in Figure 3A with an additional weight-dependent rule in which the amount of LTP was inversely related to synaptic size (Figure 4D). The ability of stronger inhibition to confer a competitive advantage to P1 was maintained.

### Influence of inhibitory neuron gain on STDP

**Figure 6:**
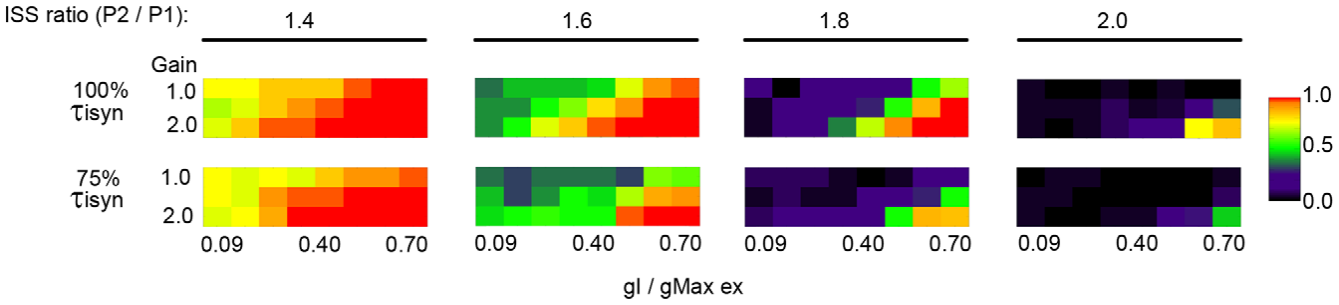
An increase in gain can compensate for the decrease in the decay time-constant of synaptic inhibition. The fraction of trials in which P1 out-competed P2 was diminished when the decay time-constant of synaptic inhibition (τ_isyn_) was decreased to 75% of the original value of 5.75 ms. However, an increase in gain, implemented as an increase in number of inhibitory presynaptic spikes, increased the fraction of trials in which P1 out-competed P2.

Our whole-cell recordings from pyramidal neurons in cortical slices revealed that while the amplitude of unitary IPSCs increased at the onset of the critical period, there was a concomitant decrease in the synaptic decay time-constant, thus the developmental change in unitary synaptic charge does not scale equally with amplitude. When the 25% decrease in synaptic decay time-constant that we experimentally observed was implemented in the simulation, we found that the effectiveness of increased inhibition in ensuring that P1 out-competed P2 was diminished (Figure 6). We hypothesized that the developmental increase in gain of spike output in GABAergic interneurons that we experimentally observed could compensate for the decrease in the synaptic decay time-constant. We tested this hypothesis by first demonstrating that an increase in gain of spike output increased the probability of P1 out-competing P2 across a range of initial synaptic strength values (Figure 6). In this case the synaptic inhibitory decay time-constant was set to the original value of 5.75 ms, and increased gain was implemented as the probability of a given inhibitory input spiking twice. For example, a gain of 1.0 corresponded to a given presynaptic input spiking only once, a gain of 1.5 corresponded to a 50% probability that a given presynaptic would generate a second spike, and a gain of 2 signified that a presynaptic input would spike twice. Pv+ interneuron-to-pyramidal synapses display short-term depression in which the amplitude of the second IPSC is 10–30% reduced compared to the first IPSC (Figure S5). To account for short-term depression in our simulation, the amplitude of the conductance on the second spike was reduced by 20%. We found that increasing spike output gain increased the probability of P1 out-competing P2. Next, the gain was increased at the same time the synaptic decay time-constant was reduced to 75% of the original value. We found that an increase in gain could indeed compensate for the decreased synaptic decay time-constant.

In summary, our modeling results show that both temporal coherence and initial synaptic strength of synaptic inputs can confer a competitive advantage at convergent pathways modifiable by STDP, but synaptic inhibition can constrain STDP to favor temporally coherent inputs at the expense of inputs with stronger initial synaptic strength. These results have implications for ocular dominance plasticity, particularly for class 2/3 neurons. Given that OD plasticity involves correlation-based Hebbian mechanisms [25], a prerequisite for a binocular neuron to shift its ocular dominance towards the open eye is that its spike times must correlate with the open eye pathway. For a class 2/3 neuron during contralateral eye closure, this means that the weaker but more coherent inputs of the open eye pathway must increase the correlation of their spike times with the spike times of the postsynaptic neuron. Studies using single unit recordings generally classify cells as 2/3 if the contralateral drive is 1.5 fold or greater than the ipsilateral drive [50], thus the ISS_P2/P1_ ratios we used are well within the range of experimental observations, and the effects that we observe on STDP may also apply to cells that are borderline class 4 cells. Our modeling results demonstrate that the maturation of GABAergic inhibition can constrain STDP so that the spike output of class 2/3 neurons, which are dominated by the contralateral eye at the time of its closure, become increasingly correlated with the ipsilateral, open-eye input. Our simulation further shows that inhibition mediates such an effect by reducing the synaptic efficacy of the less coherent, even though stronger, inputs. Therefore, a prediction from our model is that maturation of GABAergic inhibition must be sufficiently strong to more potently decrease synaptic efficacy at the peak versus prior to the onset of the critical period of OD plasticity.

### Maturation of visual cortical synaptic inhibition reduces synaptic efficacy

We used a visual cortical slice preparation to examine whether the maturation of GABAergic inhibition reduces synaptic efficacy, and whether this effect correlates with the onset of OD plasticity. Postsynaptic responses in layer 2/3 pyramidal neurons were recorded following stimulation of layer 4, which evoked a mixed excitatory-inhibitory response (Figure 2A). The time course of evoked IPSCs outlasted the EPSCs by roughly 7-fold. This was due to the slower kinetics of the GABA_A_ receptors compared to that of the AMPA receptors and to the presence of polysynaptic IPSCs.

**Figure 7:**
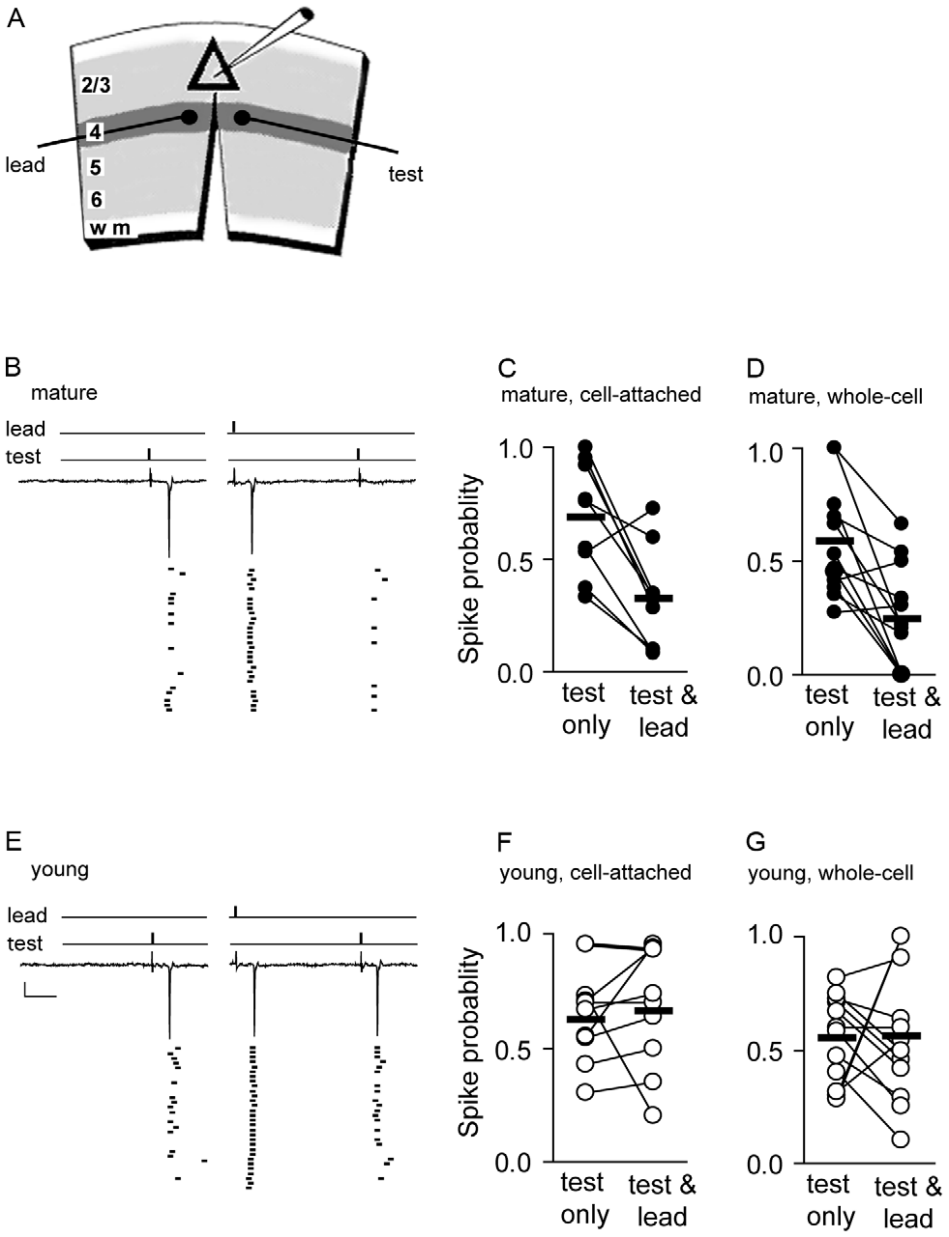
The potency of one input pathway to suppress the probability of a convergent pathway from triggering a postsynaptic spike in layer2/3 pyramidal neurons in V1 is developmentally regulated. (A) An experimental paradigm in visual cortical slices to examine the interaction of two convergent input pathways to drive postsynaptic spiking at layer 4→layer 2/3 connections. Triangle represents a layer2/3 pyramidal soma. Field stimulation sites for the lead and test pathways are depicted. The slice is cut between the stimulation sites to ensure that the two pathways are independent. The ability of P_lead_ to suppress threshold stimulation of P_test_ was assayed in both cell attached mode and whole-cell mode in mature (B–D) and young (E–G) slices in the presence of APV and CGP55845. (B,E) Example traces of cell-attached recordings with inter-leaved trials in response to pathway stimulation. Stimulus artifacts were clipped. Action potentials were detected as a capacitive transient, approximately 500–600 pA in amplitude. Raster plots of spike events are aligned to onset of P_lead_ stimulation. 30 trials are displayed, 30–60 trails were collected for each cell. Spike probability was assayed from 2 to 10 ms following stimulation of the P_test_ during the Lead-Test trial,scale bar: 100 pA,10 ms. (B–D) Spike probabilities of each cell during Test Only and Test-Lead trial stimulations are shown for cell-attached (C; n = 9), and whole-cell (D; n = 12) recordings from mature visual cortical slices (P26–30), mean probability (black bar). (E–G) Same as in b-d except recordings were done in young slices (P16–18). Cell-attached, n = 9, whole-cell, n = 12. Note that P_lead_ potently suppressed P_test_ from driving postsynaptic spiking in mature but not young slices.

Previous studies have shown that stimulus-evoked inhibition can reduce the synaptic efficacy of asynchronous EPSPs for up to 30–50 ms [30], [51], a time course that matches the duration of the evoked GABA_A_ current measured here. We used a two-pathway stimulation paradigm to examine the effect of synaptic inhibition on synaptic efficacy at layer 4 to layer 2/3 connections (Figure 7A). The stimulation intensity of both pathways was normalized to spike threshold in layer 2/3 pyramidal neurons to facilitate comparison across slices and animals. A test pathway (P_test_) was stimulated at threshold intensity such that an action potential in a layer 2/3 pyramidal neuron was generated with a probability of approximately 0.5 (see methods). The ability of P_test_ to trigger a postsynatpic action potential was then challenged by stimulating a leading pathway (P_lead_) 40 ms earlier. The stimulation intensity of the P_lead_ was set such that an action potential in the postsynaptic pyramidal neuron was triggered with >0.9 probability. P_test_ and P_lead_ were verified to be independent pathways to avoid short-term plasticity such as synaptic depression. The use of the 40 ms interval between the two stimuli ensured that postsynaptic spikes evoked by P_lead_ stimulation did not over lap with the postsynaptic responses evoked by P_test_ stimulation. Trials in which only P_test_ (Test Only) was stimulated were interleaved with those in which P_lead_ and P_test_ were sequentially stimulated (Lead-Test).

The first experiment was done in cell-attached mode, with an intact intracellular chloride gradient. We found that at the peak of the critical period (P26–30), P_lead_ stimulation reduced the spike probability (ρ) of layer 2/3 pyramidal neurons in response to P_test_ stimulation by 37+/−0.09% (lead-test: ρ = 0.32+/−0.08, test alone: ρ = 0.69+/−0.08, n = 9, Wilcoxon signed rank, p<0.02, Figure 7B,C). Prior to the onset of the critical period (P16–18), however, there was little if any effect of P_lead_ stimulation on spike probability of layer 2/3 pyramidal neurons triggered by P_test_ stimulation (lead-test: ρ = 0.66+/−0.09, test alone: ρ = 0.62+/−0.06, mean difference: Δ+0.04+/−0.08, n = 9, Wilcoxon signed rank, p = 0.25, Figure 7E,F).

We then repeated the above stimulation protocol using whole-cell recordings of layer 2/3 pyramidal neurons. Similar to cell-attached recording, spike probability in layer 2/3 pyramidal neurons in response to P_test_ stimulation was significantly reduced by P_lead_ stimulation at the peak of the critical period (lead-test: ρ = 0.25+/−0.07, test alone: ρ = 0.58+/−0.07 mean difference: Δ−0.034+/−0.08, n = 12, Wilcoxon signed rank, p<0.003, Figure 7D), while no such effect of P_lead_ stimulation was found prior to the onset of the critical period (lead-test: ρ = 0.56+/−0.08, test alone: ρ = 0.55+/−0.06, mean difference: Δ+0.01+/−0.11, n = 12, Wilcoxon signed rank, p = 0.58, Figure 7G).

**Figure 8:**
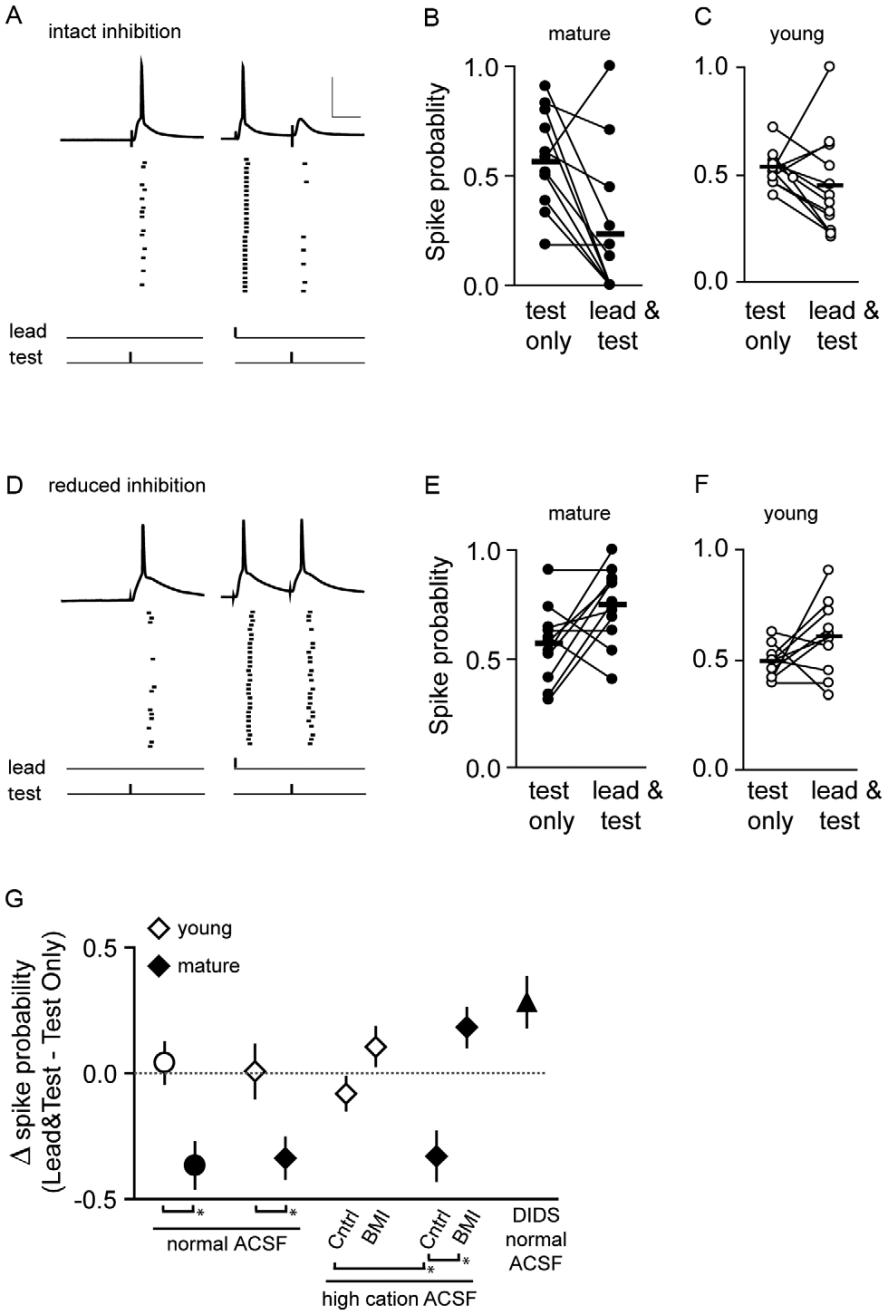
Reduction of synaptic efficacy of P_test_ by P_lead_ stimulation is mediated by synaptic inhibition. Whole-cell recording of spike probability of layer 2/3 pyramidal neurons in V1 in response to the Test Only and Lead-Test pathway stimulation paradigm (Figure 7A); bathing media contained high cations (3 mM CaCl_2_ and 3 mM MgSO_4_). (A) Example traces of two inter-leaved trials in response to pathway stimulation in mature slices with intact inhibition. Raster plots of spike events are shown below the traces. Spike probabilities of individual pyramidal neurons in response to Test Only and Lead-Test pathway stimulation with intact inhibition are then plotted for the mature (B) and young (C) age group. (D–F) Same as in A–C except inhibition was reduced with the application of 3 µM BMI. Scale bar: 50 mV, 20 ms. (G) Summary plot of the average differences in spike probability of layer 2/3 pyramidal neurons in response to Test Only and Lead-Test stimulation. Circles represent cell-attached recordings, diamonds and triangle represent whole-cell recordings. GABA_B_ and NMDA-mediated currents were blocked. Asterisks denote a significance effect of treatment (p<0.05), determined by ANOVA analysis.

To examine whether the reduction in synaptic efficacy of P_test_ was mediated by synaptic inhibition, we repeated the whole-cell experiment in a bathing solution containing 3 mM divalent cations and 3 µM bicuculline methiodine (BMI). This resulted in an 80% block of synaptically evoked GABA_A_ current (data not shown) without inducing epileptic activity in cortical slice. In the presence of raised cation concentration but in the absence of BMI, spike probability in layer 2/3 pyramidal neurons in response to P_test_ stimulation was significantly reduced by P_lead_ stimulation at the peak of the critical period (lead-test: ρ = 0.23±0.10, test only: ρ = 0.56±0.07, mean difference: Δ−33+/−0.10, n = 12, Figure 8A,B), similar to the above results of Figure 7. The effect of P_lead_ stimulation was blocked in the presence of BMI (lead-test: ρ = 0.75±0.05, test only: ρ = 0.56±0.05, n = 11, mean difference: Δ+0.18±0.08, Figure 8E). In contrast, prior to the onset of the critical period, BMI had little impact on the ability of P_lead_ to reduce spike probability in response to P_test_ stimulation (no BMI, lead-test: ρ = 0.45±0.07, test alone: ρ = 0.53±0.02, mean difference: Δ−0.08±0.07, n = 12, Figure 8C; BMI, lead-test: ρ = 0.60±0.50, test alone: ρ = 0.49±0.02, mean difference: Δ+0.11±0.06, n = 10, Figure 8F). A 2-way ANOVA was performed to determine whether the age-dependent effect of GABA_A_ blockade was significant. The change in spike probability (lead-test – test only) for layer 2/3 pyramidal cells was compared across treatment groups (Figure 8G). The interaction between age and BMI treatment was significant (p<0.05), and the effect of BMI on spike probability was significant (p<0.001). Subsequent pairwise comparisons using the Holm-Sidak method revealed a significant difference between BMI treated and control cells in the mature age group, but not in the young age group. We conclude that there is a developmental increase in the ability of synaptic inhibition to decrease synaptic efficacy during the critical period in mouse visual cortex.

We noted that reduced GABA_A_ conductance revealed the presence of a slight summation among inputs. This effect was also seen by Mittman et. al. (2005); given the rapid kinetics of AMPA receptors, this effect was unlikely due to synaptic conductance evoked by stimulation of P_lead_. The effect could not be explained by a change in input resistance (young: control, 262.38±3.89, BMI, 261.62±4.96; mature: control, 132.50±3.38, BMI, 135.57±2.34 MΩ), suggesting that the slight summation observed in the condition of 80% GABA_A_ block may be due to a voltage-dependent persistent sodium conductance induced by stimulation of P_lead_ [52].

Further evidence for the role of chloride conductance in mediating the reduction in spike probability at the peak of the critical period was obtained by using DIDS-fluoride in the recording pipette to block anionic conductances, including GABA_A_ [53]. Because the drug was in the pipette, the blockade was specific to the recorded cell. Spike probability in response to P_test_ stimulation was significantly reduced by P_lead_ stimulation (lead-test: ρ = 0.61±0.07, test alone: ρ = 0.32±0.06, mean difference: Δ+0.28+/−0.01, n = 8, Wilcoxon signed rank, p<0.03, Figure 8G). In this case, there was a 2.8 fold change in input resistance that likely contributed to summation (break in: 154±37, stable: 348±50 MΩ). The change in spike probability due to stimulation of P_lead_ for all treatments is summarized in Figure 8G.

In summary, synaptic inhibition evoked by the P_lead_ was effective in reducing synaptic efficacy of P_test_ at the peak of but not prior to the onset of the critical period of OD plasticity.

## Discussion

A significant advance towards understanding the mechanism of association-based Hebbian plasticity is the discovery of spike-timing-dependent plasticity. In addition to providing an explanation for how the strength of synaptic connections can be modified based on the causality of pre and postsynaptic spike events, STDP is inherently stabilizing. Modeling studies indicate that STDP implemented in its most basic form leads to a stable distribution of synaptic conductances, STDP forces the postsynaptic neuron into a balanced regime in which the net excitatory drive onto the postsynaptic cell remains constant: inputs that repeatedly take part in firing the postsynaptic cell are strengthened at the expense of those that do not. STDP-mediated re-organization of synaptic strengths is therefore also inherently competitive. A major issue in STDP, and stabilization of Hebbian plasticity in general, is to define the parameters and conditions that select for one group of inputs over another group. Temporal coherence among inputs has been well recognized to impact postsynaptic spiking and confers a competitive advantage during STDP-mediated re-organization of synaptic weights [21], [22]. Additionally, synaptic strength exerts a critical influence on the outcome of STDP [9], [23], [24] However, it is not clear how synaptic inhibition, central in controlling many aspects of synaptic summation and spike generation, regulates the manner in which these two input parameters direct the outcome of STDP.

Our modeling study here showed that synaptic inhibition constrained STDP to favor temporally coherent inputs at the expense of stronger, less coherent inputs. Inhibition exerted this effect by preferentially reducing the synaptic efficacy of the less coherent inputs. In visual cortical slices, we showed that STDP is expressed at postnatal ages that correspond to the peak as well as prior to the onset of the critical period; and that GABAergic inhibition more potently reduced synaptic efficacy at the former compared to the latter age. These results have implications for the role of GABAergic inhibition in visual cortical plasticity.

### Inhibition constrains STDP to favor temporal coherence over initial synaptic strength

The initial synaptic strength among converging inputs in neural circuits may be determined by genetic mechanisms and/or prior activity-dependent modifications. As an effective plasticity mechanism, STDP must maintain the capacity to modify synaptic strength, including the weakening of strong synapses, according to the on-going patterns of input activity. This is crucial in order for circuits to refine connectivity based on experience. By using a simple integrate-and-fire model neuron driven by two input pathways, we compared the effectiveness of temporal coherence versus initial synaptic strength in shaping the outcome of STDP. In addition, we compared the effectiveness of temporal coherence versus number of inputs within a given pathway. Here we show that the competitive advantage that temporally coherent inputs have over initially stronger inputs does not result from the intrinsic properties of STDP, but rather requires constraints by synaptic inhibition.

Synaptic inhibition potently influences input summation required for spike generation by restricting the temporal window over which inputs are able to effectively cooperate [29]–[31]. In vivo recordings in primary sensory cortex demonstrate that inhibition exerts this effect by increasing the requirement for temporal coherence among inputs to evoke spiking beyond what is set by the membrane time-constant of the postsynaptic neuron [31], [54]. The precise manner by which synaptic inhibition is recruited during sensory experience is likely influenced by many factors [34], [55], [56]. In our model therefore, we implemented synaptic inhibition using the least number of assumptions: every EPSP was followed by an IPSP with a delay ranging between 4 to 10 ms; and for a given simulation, the amplitude of inhibition was fixed. We then systematically varied the amplitude of inhibition across simulation trials. We found that, independent of STDP, an increase of inhibition reduced the synaptic efficacy of both P1 and P2 pathways, but the reduction was more profound for the less coherent, even though stronger, P2 pathway (Figure 5; compare A&B). The small difference in synaptic efficacy between the two pathways brought about by synaptic inhibition had a major impact on STDP-mediated re-organization of synaptic weights (Figure 5; compare C,E,&D,F).

Our results thus suggest that, at low levels of inhibition, strong but less coherent inputs effectively competed with weaker but more coherent inputs; the stabilizing property of STDP favors the maintenance of the existing synaptic weight distribution over updating the distribution according to novel temporal patterns of input. At higher levels of inhibition, on the other hand, the efficacy of the less coherent inputs is preferentially reduced, biasing STDP to increasingly favor the more coherent inputs. Therefore, a sufficient level of inhibition is crucial to regulate STDP such that inputs are modified according to their correlation structure, a parameter that is often controlled by peripheral sensory events [27].

### Maturation of inhibition in OD plasticity and critical period

Monocular deprivation during a critical period induces a shift in the ocular dominance (OD) of binocular neurons in V1 such that the open eye pathway dominates in driving spiking activity. The OD shift involves both a reduced drive from closed eye inputs [57], [58] and an increased drive from open eye inputs [59]. The recent finding that monocular blurring rapidly shifts OD indicates that it is the quality rather than the quantity of retinal illumination that is the key factor for OD plasticity [26]. These results suggest that it is the temporal *pattern* rather than the overall *rate* of activity that drives receptive field plasticity [27]. Accordingly, in our model we altered temporal correlation of inputs but held the overall rate of the two pathways fixed at 20 Hz.

Multiple forms of cellular plasticity, operating on different time-scales, likely mediate OD plasticity in vivo at excitatory synapses. MD has been shown to induce classic homosynaptic LTD [58], [60], modeling studies suggests that STDP may also account for some forms of OD plasticity [23]. Homeostatic mechanisms also likely contribute on a slower time-scale [61], [62]. Similarly, it appears that STDP alone cannot account for the MD-induced ocular dominance plasticity that is observed in fast-spiking interneurons [63]. In addition to STDP, simulations of interneuron plasticity must add additional rules of synaptic elimination to recapitulate experimental results. Furthermore, it was recently demonstrated that in acute slices, the polarity and magnitude of associative plasticity can be regulated by neuromodulators applied generally to the bathing perfusion, raising the possibility that under some neuromodulatory states in vivo, timing rules of STDP are not rigorously bi-directional [64]. Here we find that both layer 4→ layer 2/3 inputs and local recurrent inputs are modifiable by STDP in V1 prior to and during the critical period in mice. Our results are consistent with and extend previous work that examined local recurrent connections in rat V1 [16]. It has been demonstrated that cortical synapses can be modified by STDP rules *in vivo* in response to visual stimulation [15], [17], [18], indicating that the temporal precision required for cortical STDP is propagated to upper cortical layers during sensory experience. The current available evidence supports the view that in response to MD, the synaptic weight distribution of synapses converging onto binocular neurons is re-organized and stabilized in part by STDP. Regardless of the precise contribution of these cellular mechanisms to the OD shift itself, a prerequisite for plasticity to proceed according to Hebb's rule is that the temporally coherent open eye inputs must correlate their spike times well with those of their postsynaptic binocular neuron. Our results suggest that GABAergic inhibition is required for this prerequisite to be met.

Maturation of GABAergic inhibitory circuits has been implicated in the regulation of critical period plasticity in visual cortex [37], [38]. Particularly compelling is the finding that direct enhancement of GABAergic transmission induces precocious OD plasticity [36], [39], [40]. This result indicates that the machinery for OD plasticity is operational even before its natural onset but lies dormant, and can be triggered by the maturation of GABAergic transmission. However, the cellular and synaptic mechanisms by which GABAergic inhibition regulates OD plasticity remain elusive. It is also unclear how GABAergic inhibition is related to correlation-based plasticity mechanisms. Our modeling study now demonstrates that a sufficient level of synaptic inhibition is crucial to constrain STDP so that synaptic strengths are modified according to their correlation structures rather than their initial synaptic drive. We further provide experimental evidence that maturation of inhibition is sufficient to potently reduce synaptic efficacy during the critical period. These results are consistent with the notion that inhibition preferentially reduces the synaptic efficacy of the less coherent inputs among convergent pathways.

**Figure 9:**
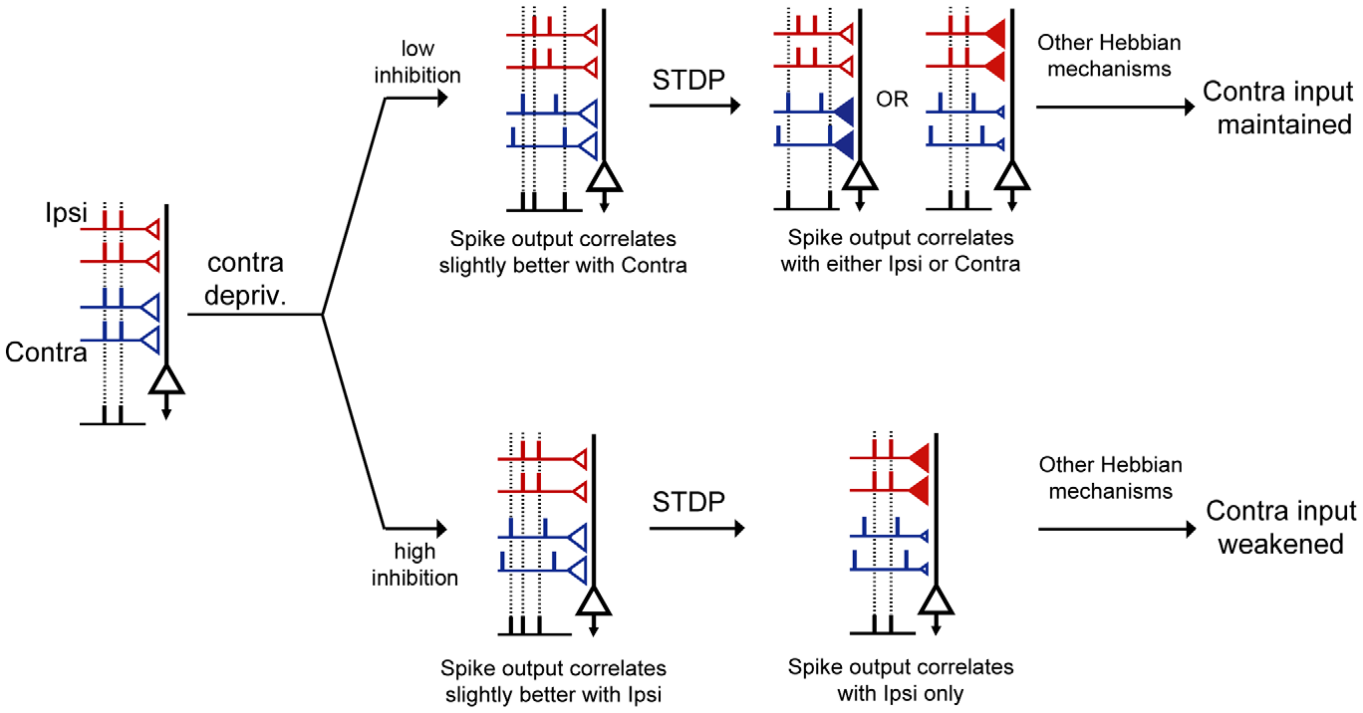
Proposed mechanism by which maturation of inhibition promotes OD shift of class 2/3 neurons in V1. Symbol designs are the same as in Figure 2. In addition, the filled red and blue triangles highlight the increase of synaptic weights. From left to right, a class 2/3 binocular neuron is driven by convergent ipsi- (red) and contralateral (blue) input pathways. The contralateral bias is indicated by the larger triangles in the blue pathway. Contralateral deprivation decreases the temporal coherence among inputs of the contralateral pathway. The effect of this altered temporal structure on the correlation between pre- and postsynaptic spike times is dependent on the level of inhibition. When inhibition is immature (low inhibition, upper row), spike output of the class 2/3 neuron correlates slightly better with the contralateral input compared to the ipsilateral input. Through STDP, either the ipsi or the contralateral pathway can increase its correlation with the postsynaptic outputs at the expense of a decreased correlation of the other pathway (STDP-mediated increases in synaptic weight are indicated by filled triangles and decreases indicated by smaller open triangles). Since P1 inputs fail to drive postsynaptic spiking, Hebbian mechanisms will not be able to weaken the contralateral deprived eye inputs and strengthen the ipsilateral open eye input. A shift in OD fails to occur. When inhibition is mature (lower row), the spike output of the class 2/3 neuron correlates slightly better with the more coherent ipsilateral, open eye input. In the presence of STDP, open-eye inputs will be able to control postsynaptic spike timing, thus, association-based Hebbian mechanisms can proceed.

Due to the dominance of crossed contralateral retinal projections, most binocular neurons in rodent V1 are characterized as class 2/3 neurons, driven strongly by contralateral input, and weakly by ispilateral eye input. Closure of the contralateral eye likely reduces the temporal coherence of inputs in this pathway compared to that of the ipsilateral, open eye pathway. Prior to the onset of OD plasticity, GABAergic inhibition is immature (Figure 9, upper row) and is ineffective in reducing the synaptic efficacy of inputs (regardless of their temporal coherence), and the temporal window of input summation is wide. The stronger contralateral inputs, though less coherent, continue to drive the postsynaptic neuron and correlate with postsynaptic spiking. Thus, at low level of inhibition, temporal coherence is not sufficient to control postsynaptic spike timing. As a consequence, correlation-based plasticity mechanisms cannot be engaged and the shift in OD fails to occur. STDP in fact disrupts Hebbian processes from selecting temporally coherent inputs. At the peak of critical period, inhibition is mature (Figure 9, lower row), which restricts the temporal window of input summation and thus preferentially reduces the efficacy of the less coherent contralateral inputs. As a consequence, the spike times of the ipsilateral pathway are better correlated with postsynaptic spike times compared to those of the contralateral pathway (Figure 9, lower row). In the presence of the STDP rule, the ipsilateral pathway consistently dominates the control of spike timing of postsynaptic neurons. Therefore, correlation-based mechanisms are able to strengthen ipsilateral inputs, weaken the contralateral inputs, and OD plasticity proceeds. By modeling specific features of temporal input structure that are disrupted during MD, we were able to characterize the effect of increasing inhibition on STDP. Other Hebbian and non-Hebbian mechanisms operating at various time scales are likely involved in mediating the loss of responsiveness of the contralateral pathway (Figure 9, far right) [55], [58], [65]. In summary, the maturation state of GABAergic inhibition likely has a major impact on whether the synaptic weight distribution can be updated to reflect to novel patterns of sensory input, including monocular deprivation at the onset of the critical period for OD plasticity.

A complete understanding of the impact that maturation of inhibition has on OD plasticity will require closer examination of interneuron binocular plasticity and initial contralateral bias during development. A recent study examining layer 2/3 interneuron calcium activity reported an initial contralateral bias in interneurons similar to that of pyramidal cells, and a delayed shift in ocular dominance relative to pyramidal cells in response to monocular deprivation. Modeling results indicated that a delayed shift of interneurons potently increased the rate of pyramidal ocular dominance plasticity [66]. If it is the case that the inhibition/excitation ratio for a given postsynaptic pyramidal cell is higher in response to contralateral stimulation compared to ipsilateral stimulation during the pre-critical period, the need for maturation of inhibition to regulate STDP as we describe may not be as strong. However, two other recent studies observe a different pattern of interneuron recruitment in which there is little or no initial contralateral bias of interneurons [63], [67]. Under conditions in which the contralateral pathway does not preferentially drive inhibition the requirement for inhibition to regulate STDP is maintained.

It has been proposed that maturation of inhibition may promote STDP to efficiently induce LTD of deprived inputs during OD plasticity [10], [68]. In this scenario, loss of responsiveness of deprived inputs is directly mediated via STDP. Furthermore, there is precedent for STDP to actively participate in the selective weakening of deprived inputs in barrel cortex [19]. Interestingly, our modeling results demonstrate that the mechanism by which increased inhibition promotes STDP-mediated LTD does not necessarily involve more LTD of the less coherent inputs due to increasing the probability that these inputs fall in the LTD portion of the STDP rule (Figure 4G,H), which is extended compared to the LTP portion [10]. Instead, we describe a set of conditions in which increased inhibition increases the relative number of coherent inputs that fall into the LTP window (Figure 4A–F). Thus, it appears that inhibition can exert a potent effect on the outcome of STDP-mediated competition by regulating synaptic efficacy.

### Diverse sources of synaptic inhibition

Synaptic inhibition is mediated by diverse types of GABAergic interneurons [69]. Our point-conductance model implies that perisomatic inhibition is a candidate source of synaptic inhibition. Consistent with this notion, parvalbumin-positive (Pv+) perisomatic GABAergic synapses structurally mature during the critical period of OD plasticity [34], and there is evidence that α1-containing GABA_A_ receptors, which are enriched at the perisomatic region, contribute to OD plasticity [40]. Here we demonstrated that on average, there was a 2-fold increase in IPSC amplitude at unitary Pv+ interneuron-to-pyramidal connections during the critical period compared to that prior to the critical period. We also found that the stimulus input/spike output curve of Pv+ interneurons matured during the critical period, raising the possibility that an increase in the gain of Pv+ interneurons may contribute to the onset of the critical period. Indeed, increasing gain or IPSC amplitude had a similar effect on STDP-mediated redistribution of synaptic weights in our simulation. Furthermore, the increase in gain was sufficient to compensate for the developmental decrease in IPSC decay time-constant.

It is important to note that in addition to spike generation, inhibition may constrain STDP by regulating the propagation of dendritic action potentials [70]. This effect is not simulated in our point conductance model. Although dendtritic-targeting interneurons also mature during postnatal development [71], whether their maturation correlates with the critical period remains to be investigated.

By modeling primary features of MD-induced alterations in temporal input structure we demonstrated that regardless of the extent to which STDP mediates the shift in ocular dominance, the potent stabilizing property of STDP can in fact disrupt ocular dominance plasticity from proceeding unless constrained by inhibition. As predicted by the model, we found that maturation of inhibition decreases synaptic efficacy at the peak of the critical period. Our results highlight the need for circuits to regulate powerful stabilizing mechanisms such as STDP in order for experience-dependent plasticity to proceed.

## Methods

### Ethics statement

All procedures were approved by CSHL IACUC.

### Slice preparation and electrophysiology

Acute cortical slices of visual cortex were prepared from C57B6 mice, age postnatal day (P)16-18 (young), or P26–30 (mature), unless otherwise noted in the text. Brain slices (300 microns thick) were cut in the coronal plane with a vibroslicer (Vibratome, St. Louis, MO) in ice-cold dissection ACSF (in mM): 212.7 sucrose, 2.5 KCl, 1.25 NaH_2_PO_4_, 3 MgSO_4_, 1 CaCl_2_, 10 D(-)-glucose, and 26 NaCHO_3_, continuously bubbled with 95%O_2_/5%CO_2_ and allowed to recover for >30 minutes in normal ACSF (in mM): 126 NaCl, 2.5 KCl, 1.25 NaH_2_PO_4_, 1 MgSO_4_, 2 CaCl_2_, 10 D(-)-glucose, and 25 NaCHO_3_ continuously bubbled with 95%O_2_/5%CO_2_ and then transferred to the recording chamber. Slices were viewed with infrared differential interference contrast optics on an upright microscope (Axioskop, Zeiss, Thornwood, NY). Slices were submerged in normal ACSF containing 50 µM APV (Tocris, Ellisville, MO) and 1 µM CGP55845 (Tocris, Ellisville, MO), except as noted, and perfused at a rate of 2–3 ml/min (33+/−1°C). Recording were made using a Multiclamp 700A amplifier (Molecular Devices, Sunnyvale, CA).

For current-clamp recordings using the STDP protocol, the intracellular solution contained (mM): 110 K-gluconate, 20 KCl, 10 HEPES; 4 MgATP, 10 phosphocreatine(Na), and 0.3 NaGTP, pH 7.3, 300 mOsm. To avoid confounding effects of inhibition when assaying plasticity at glutamatergic synapses, 10 µM picrotoxin was included in the bath perfusion to block GABA_A_ receptors. Picrotoxin is a preferred blocker over bicuculine methiodine (BMI) in synaptic plasticity assays because BMI used at concentrations sufficient to completely block GABA_A_ receptors has been shown to block SK potassium channels [72], which could potentially alter local dendritic excitability and thereby impact the induction of plasticity. For voltage-clamp recordings used in the EPSC-IPSC maximal charge assay, the intracellular solution contained (mM): 130 Cs-gluconate, 8 KCl, 10 HEPES, 10 EGTA, 10 QX-314 (Alomone, Jerusalem, Israel). For current-clamp recordings in the synaptic efficacy assay, the intracellular solution contained (mM): 135 K-gluconate, 4.3 KCl, 2 NaCl, 10 HEPES, 0.5 EGTA, 4 MgATP, 20 phosphocreatine(Na), and 0.3 NaGTP, pH 7.3, 300 mOsm. Methods currently available to block GABA_A_ receptors in cortical slices during protocols that require synaptic stimulation intensities strong enough to bring postsynaptic cells to spike threshold are limited because full blockade of GABA_A_ receptors, such as achieved with 10 µM picrotoxin in the bathing medium, will cause epileptic-like activity in response to strong synaptic stimulation. Therefore, we employed two different methods to reduce GABA_A_ receptor conductance, both have non-overlapping drawbacks. Low-concentration BMI (3 µM) in combination with raised cation concentration was previously shown to significantly reduce GABA_A_ receptor conductance without inducing epilepsy in cortical slices [16], this method was employed in Figure 8. Anion channels and pumps can be blocked intracellularly with a fluoride-based internal solution in combination with 4,4'-diisothiocyanatostilbene-2, 2'-disulfonic acid (DIDS) [73], [74]. The DIDS internal solution contained (in mM): 120 KF, 8 KCL, 10 HEPES, 10 EGTA, 1 DIDS. Whole-cell recordings pipettes had a tip resistance of 3–4 MΩ. Data were digitized at 10 kHz, filtered at 2 kHz, and analyzed with Clampfit 9 (Molecular Devices, Sunnyvale, CA). EPSP/Cs were evoked by focal extracellular stimulation (0.2 ms, 10–100 µA) with commercial bipolar electrodes (FHC, Bowdoin, ME), except in STDP protocols, a small glass bipolar electrode was used [16]. In STDP protocols, the initial EPSP slope (mV/ms) was set to be the same across ages and cells. Vertical LTP: young, 0.44+/−0.11; mature, 0.46+/−0.06 (t-test, p = 0.88). Vertical LTD: young, 0.45+/−0.05; mature, 0.34+/−0.06 (t-test, p = 0.16). Horizontal LTP: young, 0.38+/−0.08; mature, 0.42+/−0.11 (t-test, p = 0.79). Horizontal LTD: young, 0.48+/−0.08; mature, 0.35+/−0.04 (t-test, p = 0.13). Input resistance was monitored with hyperpolarizing current pulses (25 pA, 100 ms); cells were excluded if input resistance changed >30% over the entire experiment. The change in initial EPSP slope (first 2 ms) was calculated as the EPSP slope ratio by dividing the average initial slope after pairing (10–20 minutes following the EPSP and AP pairing) by the average baseline initial slope. In the synaptic efficacy paradigm, the two pathways were considered independent if EPSP slopes summed linearly [75]–[77]. P_lead_ stimulation intensity did not exceed 1.5x threshold stimulation intensity. Chemicals were purchased from Sigma-Aldrich (St. Louise, MO) except as noted. For statistical analysis, two-tailed parametric tests were used unless the data were not normally distributed. In such cases, Wilcoxon signed rank was used for paired samples, and Mann-Whitney for unpaired samples. Error is reported as ± standard error of the mean, unless noted.

### Simulation

We simulated a conductance-based integrate-and-fire model in Matlab (Mathworks, Natick, MA), using the difference equation 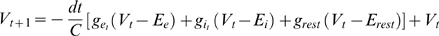, where *g*_rest_ = 12.5 nS and capacitance, C, was set to C = 0.25 nF to give a membrane time-constant of 20 ms. Ee = 0 mV, and Ei = −70 mV. When the membrane potential *V_t_* reached a threshold value of −54 mV_,_ *V* was reset to –65 mV with a refractory period of 2 ms (20 iterations or time-steps). Excitatory synaptic inputs were modeled as conductances given by the function, g(Δt) = *α*e^-Δt/τ^, where τ = 2 ms, and *α* was equal to the synaptic weight value. N = 80 excitatory synapses for all simulations. As in Song et. al. (2000), the synaptic weight value *α* was updated every iteration based on the STDP function *F*(Δ*t*):
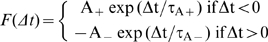
Where A+  = 0.5%* gmax_ex_, A-  = 0.45%* gmax_ex_, τ_A+_ = 20 ms, and τ_A-_ = 35 ms. gmax_ex_ was set to 15* 150 pS to give an output firing rate of 5–20 Hz for N = 80 synapses. In the weight-dependent mode shown in Figure 4, potentiation was updated as A_+_ * (1-*α*). Inhibitory conductances were also given by the function, g(Δt) = *α*e^−Δt/τ^, the decay time-constant for inhibitory synapses was initially set to τ = 5.75 ms and varied as noted in text, the value of *a* was fixed for the duration of a particular simulation, the amplitude varied between simulations as noted in the text. Excitatory presynaptic spike trains were generated in the following manner: In the case of Input Regime (I), the spike trains activating the synapses of the two sets of inputs were generated from a single Poisson process. In the case of Input Regime (II) and (III), the spike trains activating the synapses of the two sets of inputs were generated from two independent Poisson processes. The correlation coefficient between any two cells within the same pathway for the total number of spikes fired was fixed at r = 0.5 in all simulations. An additional parameter was used to define the temporal coherence (1/σ) among spike times within a given pathway. Sigma (σ) defined the temporal jitter among spike times within a given pathway, and controlled the width of the cross-correlogram peak between any two trains within the same pathway, we defined temporal coherence as the inverse of σ. Inhibitory presynaptic spike trains were implemented in a feedforward manner, every excitatory presynaptic spike was followed by an inhibitory presynaptic spike with a delay randomly ranging between 4–10 ms. The initial amplitude of inhibition was set via matching the total conductance of one inhibitory synaptic event to the total conductance of one initial excitatory event, this amplitude corresponded to 8.8% of gmax_ex_, and is indicated as 0.09 gI/gMax_ex_ in Figures 4 and 6. Simulations were run for 40–80 minutes of simulated time, except for the simulations shown in Figure 3 which were run for 12 minutes of simulated time. The Matlab M files used to generate the simulation are included in the supplement (Protocol S1).

## Supporting Information

Protocol S1 Matlab M files used to generate the IAF STDP simulation.
(0.04 MB ZIP)

Figure S1 Input/output curves used to generate Figure 2A and calculate maximal charge in Table 1. (A–D) Stimulus input/output curves in response to single pathway stimulation. (A,C) Examples of individual cells, inward EPSCs recorded at the empirically determined GABA_A_ reversal potential (gray) and outward IPSCs recorded at 0 mV (black), using a Cs-based internal solution with APV in the bath. (B,D) Absolute maximal current evoked as a function of stimulus intensity, averaged across cells (young, n = 14; mature, n = 13). Stimulation intensities used in (A): 20,30,40,50,60,70 µA, stimulation intensities used in (B): 10, 15,10,20, 30,40,50,60,70 µA. (E) Reversal potential for GABA_A_ receptor conductance was -55 mV, recorded in presence of CNQX and APV.
(0.12 MB TIF)

Figure S2 Cross-correlogram peak values of all 40 inputs for the example trials shown in Figure 3. Peak cross-correlogram values are plotted as an ascending sort for all 40 inputs of P1 (red) and P2 (blue), during the initial 50 seconds (dashed), and final 50 seconds (solid). (A–E) Peak cross-correlogram values corresponding to individual trials shown in Figure 3; in panel B, trials were sorted based on which pathway dominated at the end of the simulation. In addition to the individual trials described above, the median cross-correlaogram peak value for all trials combined for a given input regime were as follows: (A) Input Regime I, initial P1, 35.1; initial P2, 35.2; finalP1, 35.6; final P2, 35.6 spikes/second (n = 10 trials). (B) Input Regime II, trials in which Path1 dominated: initial P1, 22.4; initial P2, 14.6; final P1, 38.1; final P2, 4.5 spikes/second (n = 4). Trials in which P2 dominated: initial P1, 15.1; initial P2, 21.9; final P1, 4.4; final P2, 38.7 spikes/second (n = 6). (C) Input Regime III, initial P1, 25.0; initial P2, 6.8; final P1, 38.3; final P2, 4.4 spikes/second (n = 10). (D) Input Regime III, gI/gmax_ex_ = 0.264, trials in which P1 dominated: initial P1, 15.7; initial P2, 14.0; final P1, 37.7; final P2, 4.4 spikes/second (n = 9). Trials in which P2 dominated: initial P1, 9.8; initial P2, 17.1; final P1, 4.4; final P2, 22.1 spikes/second (n = 11). (E) Input Regime III, gI/gmax_ex_ = 0.792, initial P1, 5.9; initial P2, 5.1; final P1, 32.6; final P2, 4.4 spikes/second (n = 11).
(0.45 MB DOC)

Figure S3 The effect of increasing inhibition on pathway dominance was tested across a range of whole-cell capacitance (c) values. In all cases, stronger inhibition increased the probability of P1 to out-compete P2.
(0.16 MB TIF)

Figure S4 Synaptic efficacy of P1 and P2 for Input Regimes II and III. As in Figure 5, the relative number of presynaptic spikes that preceded post synaptic spike events was counted in 1 millisecond time bins and plotted as a function of time preceding the post synaptic spike in the absence (A,B) and the presence of the STDP rule (C–F). P1, red; P2, blue. (G–H) The relative number of presynaptic spikes that followed postsynaptic spike events was calculated as in (A–B), except that 1 ms bin counts were made for a time window of 20 ms following postsynaptic spikes. The example shown for Input Regime II is a case in which P2 out-competed P1, which occurred in half of the trials. For Input Regime III, P1 out-competed P2 in all trials.
(0.21 MB TIF)

Figure S5 Short-term dynamics of unitary IPSCs at mature Pv+ interneuron to pyramidal synapses. As expected from previous reports [78], IPSCs were depressing in response to 20 Hz stimulation, and depression was less pronounced at higher stimulation frequencies [79]. The average paired pulse ratio (IPSC_amplitude 2_/IPSC_amplitude 1_) during the critical period (age p21-27) at 20 Hz stimulation was 0.71+/−0.05 (n = 6), and at 100 Hz stimulation was 0.89+/−0.11 (n = 7). Left, example cell pair at 20 Hz stimulation, average of 15 traces. Right, example cell pair at 100 Hz stimulation, average of 5 traces.
(0.04 MB TIF)
