# Supplementary figures and images for: Maturation of GABAergic Inhibition Promotes Strengthening of Temporally Coherent Inputs among Convergent Pathways
Source: PLoS Comput Biol. 2010 Jun 3;6(6):e1000797. doi: 10.1371/journal.pcbi.1000797 (PMC2880567; doi:10.1371/journal.pcbi.1000797)

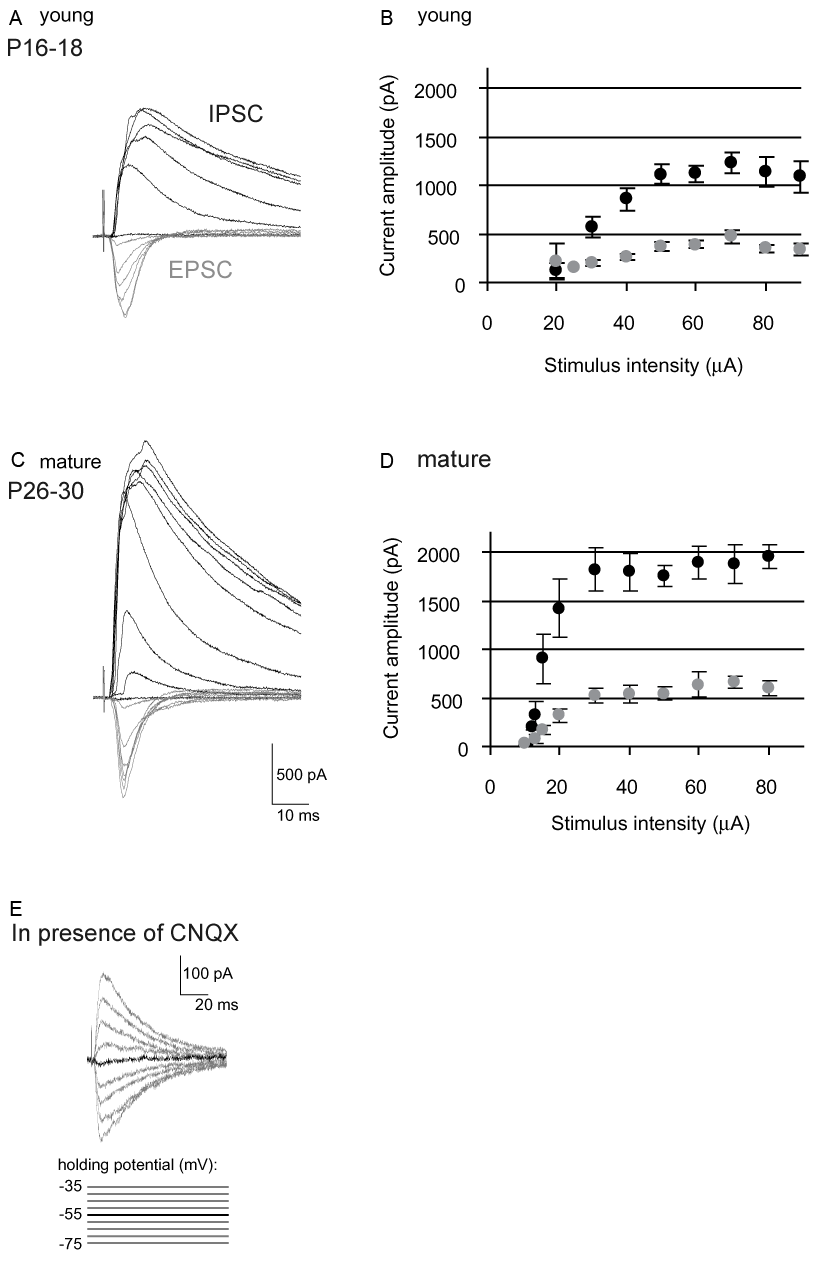

Supplement: Figure S1 — Input/output curves used to generate Figure 2A and calculate maximal charge in Table 1. (A–D) Stimulus input/output curves in response to single pathway stimulation. (A,C) Examples of individual cells, inward EPSCs recorded at the empirically determined GABAA reversal potential (gray) and outward IPSCs recorded at 0 mV (black), using a Cs-based internal solution with APV in the bath. (B,D) Absolute maximal current evoked as a function of stimulus intensity, averaged across cells (young, n = 14; mature, n = 13). Stimulation intensities used in (A): 20,30,40,50,60,70 µA, stimulation intensities used in (B): 10, 15,10,20, 30,40,50,60,70 µA. (E) Reversal potential for GABAA receptor conductance was -55 mV, recorded in presence of CNQX and APV. (0.12 MB TIF) [file pcbi.1000797.s002.tif]

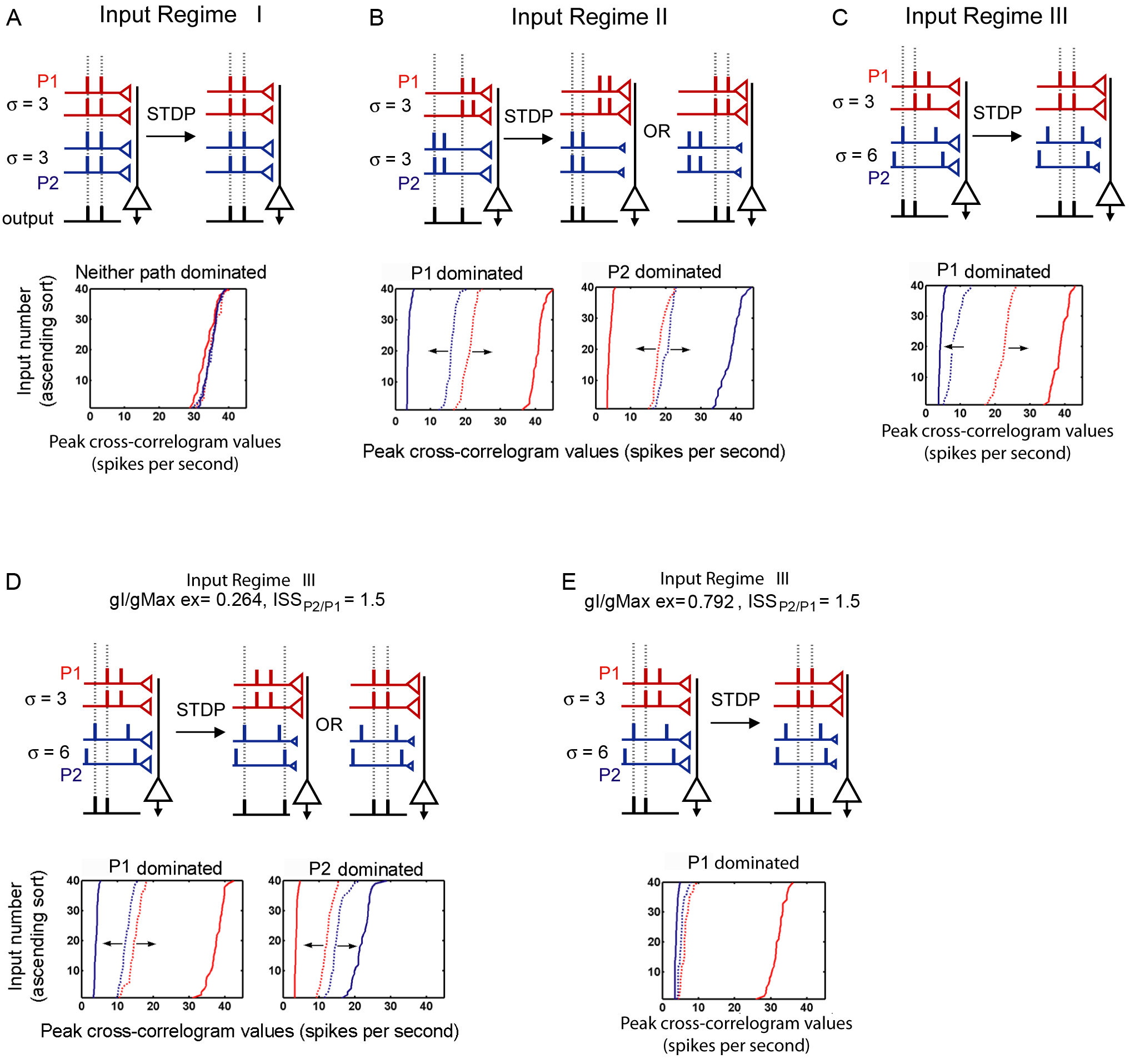

Supplement: Figure S2 — Cross-correlogram peak values of all 40 inputs for the example trials shown in Figure 3. Peak cross-correlogram values are plotted as an ascending sort for all 40 inputs of P1 (red) and P2 (blue), during the initial 50 seconds (dashed), and final 50 seconds (solid). (A–E) Peak cross-correlogram values corresponding to individual trials shown in Figure 3; in panel B, trials were sorted based on which pathway dominated at the end of the simulation. In addition to the individual trials described above, the median cross-correlaogram peak value for all trials combined for a given input regime were as follows: (A) Input Regime I, initial P1, 35.1; initial P2, 35.2; finalP1, 35.6; final P2, 35.6 spikes/second (n = 10 trials). (B) Input Regime II, trials in which Path1 dominated: initial P1, 22.4; initial P2, 14.6; final P1, 38.1; final P2, 4.5 spikes/second (n = 4). Trials in which P2 dominated: initial P1, 15.1; initial P2, 21.9; final P1, 4.4; final P2, 38.7 spikes/second (n = 6). (C) Input Regime III, initial P1, 25.0; initial P2, 6.8; final P1, 38.3; final P2, 4.4 spikes/second (n = 10). (D) Input Regime III, gI/gmaxex = 0.264, trials in which P1 dominated: initial P1, 15.7; initial P2, 14.0; final P1, 37.7; final P2, 4.4 spikes/second (n = 9). Trials in which P2 dominated: initial P1, 9.8; initial P2, 17.1; final P1, 4.4; final P2, 22.1 spikes/second (n = 11). (E) Input Regime III, gI/gmaxex = 0.792, initial P1, 5.9; initial P2, 5.1; final P1, 32.6; final P2, 4.4 spikes/second (n = 11). (0.45 MB DOC) [file pcbi.1000797.s003.tif]

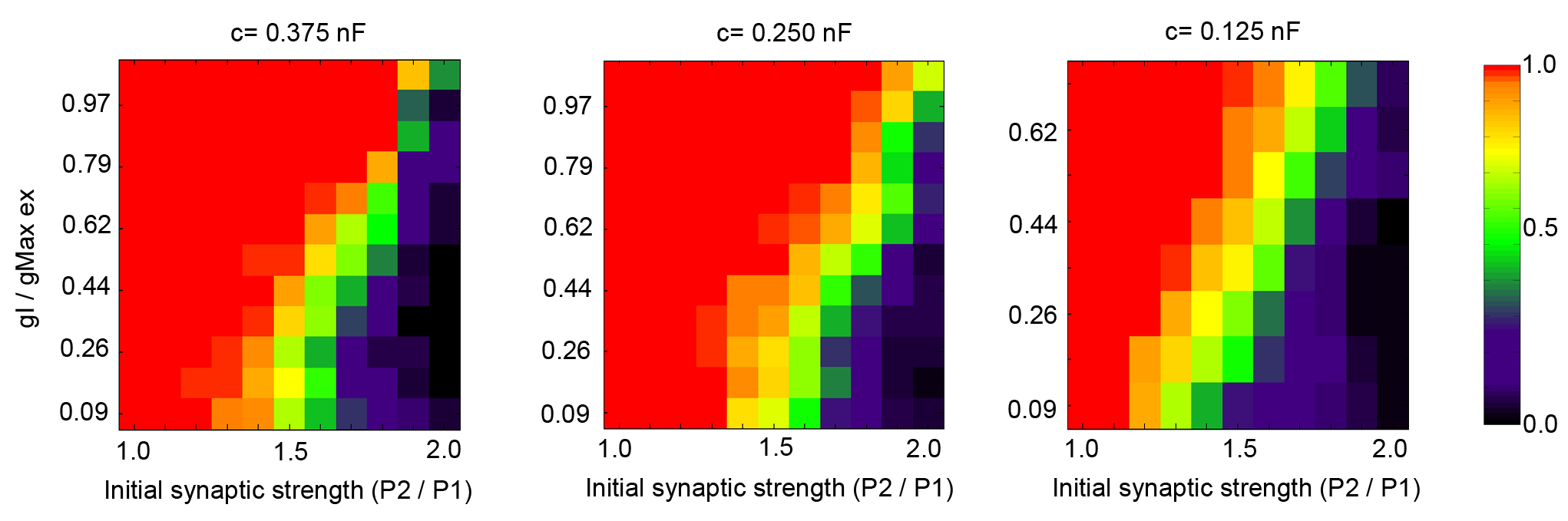

Supplement: Figure S3 — The effect of increasing inhibition on pathway dominance was tested across a range of whole-cell capacitance (c) values. In all cases, stronger inhibition increased the probability of P1 to out-compete P2. (0.16 MB TIF) [file pcbi.1000797.s004.tif]

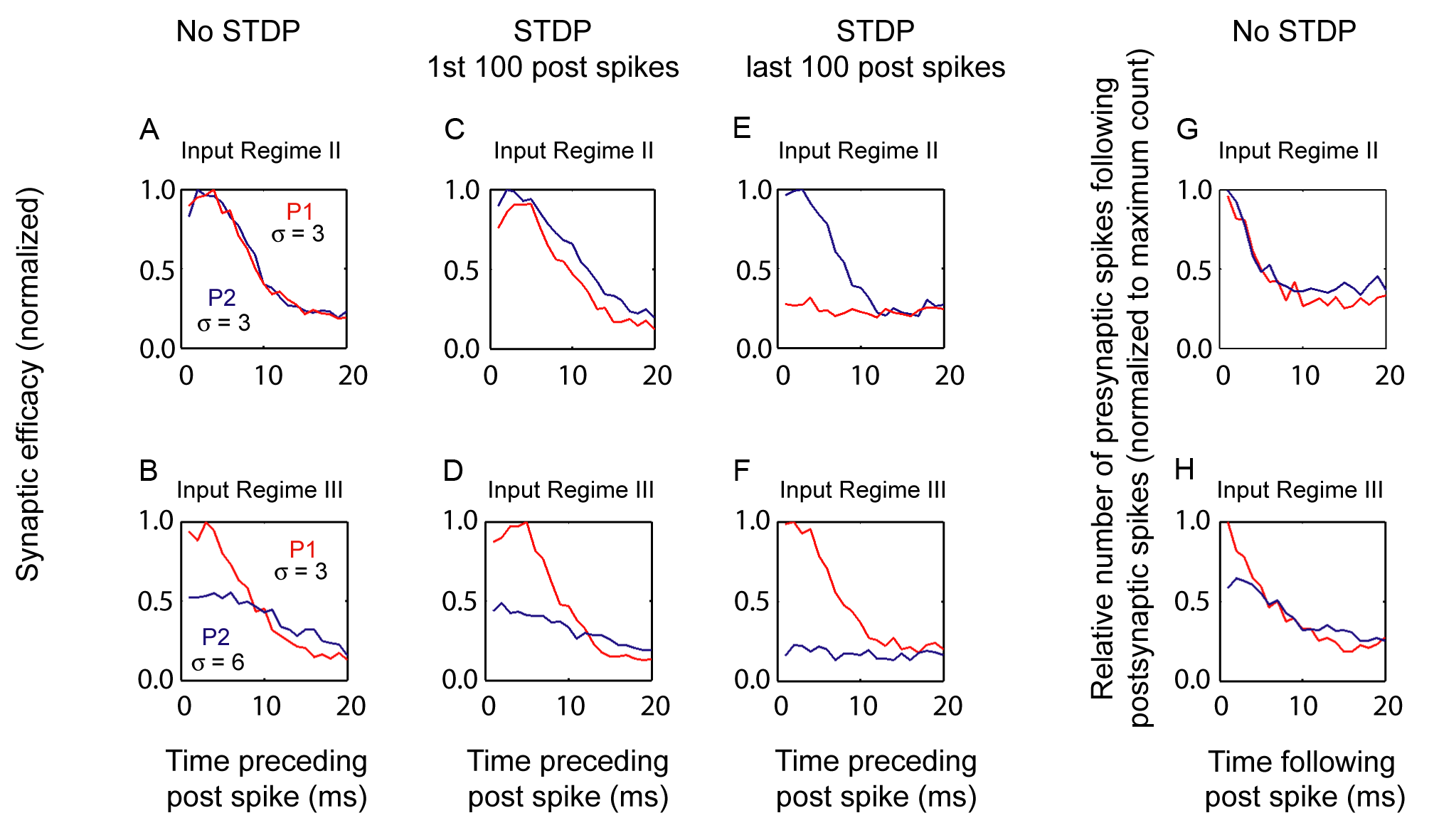

Supplement: Figure S4 — Synaptic efficacy of P1 and P2 for Input Regimes II and III. As in Figure 5, the relative number of presynaptic spikes that preceded post synaptic spike events was counted in 1 millisecond time bins and plotted as a function of time preceding the post synaptic spike in the absence (A,B) and the presence of the STDP rule (C–F). P1, red; P2, blue. (G–H) The relative number of presynaptic spikes that followed postsynaptic spike events was calculated as in (A–B), except that 1 ms bin counts were made for a time window of 20 ms following postsynaptic spikes. The example shown for Input Regime II is a case in which P2 out-competed P1, which occurred in half of the trials. For Input Regime III, P1 out-competed P2 in all trials. (0.21 MB TIF) [file pcbi.1000797.s005.tif]

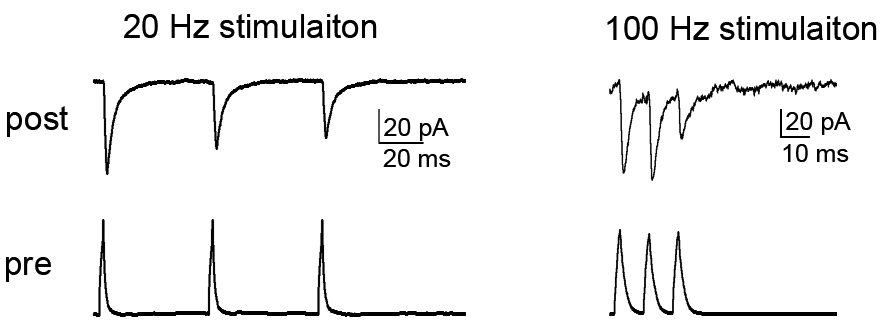

Supplement: Figure S5 — Short-term dynamics of unitary IPSCs at mature Pv+ interneuron to pyramidal synapses. As expected from previous reports [78], IPSCs were depressing in response to 20 Hz stimulation, and depression was less pronounced at higher stimulation frequencies [79]. The average paired pulse ratio (IPSCamplitude 2/IPSCamplitude 1) during the critical period (age p21-27) at 20 Hz stimulation was 0.71+/−0.05 (n = 6), and at 100 Hz stimulation was 0.89+/−0.11 (n = 7). Left, example cell pair at 20 Hz stimulation, average of 15 traces. Right, example cell pair at 100 Hz stimulation, average of 5 traces. (0.04 MB TIF) [file pcbi.1000797.s006.tif]
